# Supplementary material for: Polyketide synthase gene clusters in the ericoid mycorrhizal fungus Oidiodendron maius: genome-wide prediction, expression profiling, and functional characterization of knockout mutants
Source: Front Plant Sci. 2026 Sep 16;17:1924430. doi: 10.3389/fpls.2026.1924430 (PMC13623903; doi:10.3389/fpls.2026.1924430)
Supplement: Supplementary file 1 [file SupplementaryFile1.zip › Supplementary Materials/Supplementary Folder 1.zip!/Oidma1_AssemblyScaffolds_Repeatmasked/knownclusterblast/cluster46/jgi.p_Oidma1_60571_mibig_hits.html]

| MIBiG Protein | Description | MIBiG Cluster | MiBiG Product | % ID | % Coverage | BLAST Score | E-value |
| --- | --- | --- | --- | --- | --- | --- | --- |
| ctg1\_orf003 |  | BGC0000334 | Cyclosporin\_biosynthetic\_gene\_cluster | 31 | 94.3396226415 | 654 | 3.7e-187 |
| ADM34138.1 | non-ribosomal\_peptide\_synthetase | BGC0001084 | Notoamide\_/\_stephacidin\_biosynthetic\_gene\_cluster | 30 | 100.130123617 | 634 | 2.3e-181 |
| EPE34341.1 | Acetyl-CoA\_synthetase-like\_protein | BGC0001035 | Pneumocandin\_biosynthetic\_gene\_cluster | 29 | 100.780741705 | 634 | 3.1e-181 |
| AGC83576.1 | NRPS | BGC0000818 | Notoamide\_biosynthetic\_gene\_cluster | 30 | 99.0891346779 | 624 | 4.1e-178 |
| OAQ83772.1 | nonribosomal\_peptide\_synthase | BGC0001358 | Leucinostatins\_biosynthetic\_gene\_cluster | 31 | 96.6818477554 | 623 | 5.4e-178 |
| CBF87069.1 | nonribosomal\_peptide\_synthase,\_putative\_(Eurofung) | BGC0001290 | Emericellamide\_A\_/\_emericellamide\_B\_biosynthetic\_gene\_cluster | 31 | 93.9492517892 | 619 | 1.3e-176 |
| AFT91378.1 | EcdA | BGC0000338 | Echinocandin\_B\_biosynthetic\_gene\_cluster | 31 | 94.5348080677 | 618 | 2.3e-176 |
| BAN91483.1 | putative\_Aculeacin\_A\_nonribosomal\_peptide\_synthase | BGC0001220 | Aculeacin\_A\_biosynthetic\_gene\_cluster | 31 | 101.431359792 | 616 | 1.1e-175 |
| AAF01762.1 | AM-toxin\_synthetase | BGC0001261 | AM-toxin\_biosynthetic\_gene\_cluster | 30 | 97.7228366949 | 611 | 2.8e-174 |
| CRG85572.1 | nonribosomal\_peptide\_synthase,\_putative | BGC0001402 | Cyclochlorotine\_biosynthetic\_gene\_cluster | 28 | 99.3493819128 | 607 | 3.1e-173 |
| BAH23995.1 | nonribosomal\_peptide\_synthetase | BGC0000356 | Fumitremorgin\_biosynthetic\_gene\_cluster | 30 | 99.9349381913 | 606 | 1.2e-172 |
| BAO73252.1 | non-ribosomal\_peptide\_synthetase | BGC0000456 | Verlamelin\_biosynthetic\_gene\_cluster | 29 | 94.9902407287 | 598 | 1.9e-170 |
| AMM63162.1 | AniA | BGC0001371 | Echinocandin\_B\_biosynthetic\_gene\_cluster | 30 | 94.7950553025 | 598 | 2.4e-170 |
| EAL89049.1 | nonribosomal\_peptide\_synthase,\_putative | BGC0000355 | Fumiquinazolines\_biosynthetic\_gene\_cluster | 28 | 102.01691607 | 589 | 8.7e-168 |
| CAP96445.1 |  | BGC0000420 | Roquefortine\_C\_/\_meleagrin\_biosynthetic\_gene\_cluster | 28 | 102.01691607 | 541 | 2.7e-153 |
| EFY94500.1 | non-ribosomal\_peptide\_synthetase | BGC0000337 | Destruxins\_biosynthetic\_gene\_cluster | 30 | 81.522446324 | 538 | 3e-152 |
| AGQ43600.1 | HC-toxin\_synthetase | BGC0001166 | HC-toxin\_biosynthetic\_gene\_cluster | 31 | 87.4430709174 | 525 | 1.5e-148 |
| AAA33023.2 | HC-toxin\_synthetase | BGC0000370 | HC-toxin\_biosynthetic\_gene\_cluster | 29 | 98.6337020169 | 524 | 3.4e-148 |
| ADY16697.1 | TqaA | BGC0001142 | Tryptoquialanine\_biosynthetic\_gene\_cluster | 27 | 102.732595966 | 516 | 9.3e-146 |
| XP\_001227984.1 | hypothetical\_protein | BGC0001240 | Serinocyclin\_A\_/\_serinocyclin\_B\_biosynthetic\_gene\_cluster | 29 | 95.5107351984 | 506 | 9.7e-143 |
| AGA37267.1 | NRPS | BGC0000816 | Malbrancheamide\_biosynthetic\_gene\_cluster | 32 | 69.0956408588 | 487 | 4.6e-137 |
| EAW16180.1 | nonribosomal\_peptide\_synthase,\_putative | BGC0000293 | Acetylaszonalenin\_biosynthetic\_gene\_cluster | 32 | 70.3968770332 | 486 | 1e-136 |
| ACZ66258.1 | APS1 | BGC0000304 | Apicidin\_biosynthetic\_gene\_cluster | 26 | 104.879635654 | 467 | 8.5e-131 |
| ABB90279.1 | non-ribosomal\_peptide\_synthetase | BGC0001057 | Zearalenone\_biosynthetic\_gene\_cluster | 30 | 70.2667534157 | 464 | 5.5e-130 |
| ABM91454.1 | lysergyl\_peptide\_synthetase\_LpsB | BGC0000348 | Ergovaline\_biosynthetic\_gene\_cluster | 28 | 79.440468445 | 464 | 5.5e-130 |
| CBF76038.1 | nonribosomal\_peptide\_synthase,\_putative\_(Eurofung) | BGC0001399 | Fellutamide\_B\_biosynthetic\_gene\_cluster | 30 | 71.5679895901 | 463 | 1.2e-129 |
| CAP93139.1 |  | BGC0000357 | Fungisporin\_biosynthetic\_gene\_cluster | 30 | 71.5029277814 | 458 | 2.3e-128 |
| AFN69082.1 | nonribosomal\_peptide\_synthetase\_6 | BGC0001249 | Dimethylcoprogen\_biosynthetic\_gene\_cluster | 30 | 65.3871177619 | 447 | 6.9e-125 |
| ctg1\_orf000000 |  | BGC0000901 | Ferrichrome\_biosynthetic\_gene\_cluster | 28 | 73.1294729993 | 427 | 5.7e-119 |
| ACI30655.1 | BEAS\_beauvericin\_nonribosomal\_cyclodepsipeptide\_synthetase | BGC0000313 | Beauvericin\_biosynthetic\_gene\_cluster | 27 | 97.2023422251 | 435 | 2.7e-121 |
| AGA37269.1 | NRPS | BGC0000819 | Paraherquamide\_biosynthetic\_gene\_cluster | 28 | 88.0286271958 | 423 | 8.2e-118 |
| ACR78148.1 | BSLS | BGC0000312 | Bassianolide\_biosynthetic\_gene\_cluster | 30 | 71.9583604424 | 423 | 1.4e-117 |
| ADY16689.1 | TqaB | BGC0001142 | Tryptoquialanine\_biosynthetic\_gene\_cluster | 29 | 72.8692257645 | 418 | 2.6e-116 |
| EAL89046.1 | nonribosomal\_peptide\_synthase,\_putative | BGC0000355 | Fumiquinazolines\_biosynthetic\_gene\_cluster | 30 | 72.6740403383 | 410 | 1.2e-113 |
| CAA79245.2 | enniatin\_synthetase | BGC0000342 | Enniatin\_biosynthetic\_gene\_cluster | 29 | 70.3968770332 | 401 | 5.7e-111 |
| CAI59267.1 | peptide\_synthetase\_3 | BGC0001263\_c2 | Ergometrine\_biosynthetic\_gene\_cluster | 29 | 67.8594664932 | 384 | 4.2e-106 |
| ACJ04424.1 | aureobasidin\_A1\_biosynthesis\_complex | BGC0000307 | Aureobasidin\_A1\_biosynthetic\_gene\_cluster | 31 | 58.1001951854 | 374 | 4.4e-103 |
| AHZ34243.1 | CipF | BGC0001389 | Cichopeptin\_biosynthetic\_gene\_cluster | 27 | 104.749512036 | 374 | 5.7e-103 |
| AFJ14794.1 | PlpE | BGC0000403 | Pelgipeptin\_biosynthetic\_gene\_cluster | 26 | 97.7878985036 | 370 | 8.3e-102 |
| CAQ71829.1 | non\_ribosomal\_peptide\_synthase,\_antibiotic\_synthesis;\_contains\_4\_condensation\_domains,\_3\_AMP-acid\_ligases\_II\_domains,\_3\_PP-binding,\_Phosphopantetheine\_attachment\_site\_and\_a\_putative\_thioesterase\_domain | BGC0001189 | Taiwachelin\_biosynthetic\_gene\_cluster | 26 | 104.229017567 | 368 | 5.4e-101 |
| ALK27914.1 | non-ribosomal\_peptide\_synthase | BGC0001233 | Feglymycin\_biosynthetic\_gene\_cluster | 27 | 90.2407286923 | 365 | 2.7e-100 |
| AAF00961.1 | McyB | BGC0001017 | Microcystin\_biosynthetic\_gene\_cluster | 24 | 101.886792453 | 365 | 3.5e-100 |
| KPN90376.1 | NupC | BGC0001416 | Nunapeptin\_/\_nunamycin\_biosynthetic\_gene\_cluster | 26 | 100.130123617 | 364 | 5.9e-100 |
| AFH75321.1 | nonribosomal\_peptide\_synthetase | BGC0000425 | Sessilin\_biosynthetic\_gene\_cluster | 26 | 97.2674040338 | 360 | 1.1e-98 |
| CAG29032.1 | nonribosomal\_peptide\_synthetase\_(modules\_3\_to\_6) | BGC0001023 | Myxochromide\_biosynthetic\_gene\_cluster | 25 | 102.277163305 | 359 | 2.5e-98 |
| AGM16412.1 | paenibacterin\_synthetase\_A | BGC0000400 | Paenibacterin\_biosynthetic\_gene\_cluster | 26 | 97.918022121 | 358 | 3.3e-98 |
| CAD29798.1 | peptide\_synthetase | BGC0001015 | Microcystin\_biosynthetic\_gene\_cluster | 24 | 104.55432661 | 356 | 1.2e-97 |
| AFH75322.1 | nonribosomal\_peptide\_synthetase | BGC0000425 | Sessilin\_biosynthetic\_gene\_cluster | 26 | 94.4046844502 | 355 | 2.8e-97 |
| CAE15637.1 |  | BGC0001128 | Luminmide\_biosynthetic\_gene\_cluster | 26 | 100.325309044 | 355 | 3.6e-97 |
| AEU11006.1 | NpnB | BGC0001029 | Nostophycin\_biosynthetic\_gene\_cluster | 29 | 71.1125569291 | 355 | 3.6e-97 |
| AAO56329.1 | non-ribosomal\_peptide\_synthetase\_SyfB | BGC0000435 | Syringafactin\_biosynthetic\_gene\_cluster | 26 | 95.4456733897 | 354 | 4.7e-97 |
| KPN93065.1 | NunD | BGC0001416 | Nunapeptin\_/\_nunamycin\_biosynthetic\_gene\_cluster | 26 | 96.6167859466 | 354 | 6.1e-97 |
| ADG27359.1 | peptide\_synthetase | BGC0000296 | Actinomycin\_biosynthetic\_gene\_cluster | 27 | 98.1782693559 | 351 | 5.2e-96 |
| AHF21228.1 | TriD | BGC0000449 | Tridecaptin\_biosynthetic\_gene\_cluster | 28 | 72.9342875732 | 351 | 5.2e-96 |
| AGE25480.1 | amino\_acid\_adenylation\_protein | BGC0001208\_c2 | Poaeamide\_biosynthetic\_gene\_cluster | 27 | 95.9011060507 | 349 | 2.6e-95 |
| CCJ67638.1 | TaaC | BGC0000447 | Tolaasin\_biosynthetic\_gene\_cluster | 27 | 99.2192582954 | 347 | 5.7e-95 |
| KPN93063.1 | NupA | BGC0001416 | Nunapeptin\_/\_nunamycin\_biosynthetic\_gene\_cluster | 26 | 101.040988939 | 347 | 5.7e-95 |
| AFH75330.1 | nonribosomal\_peptide\_synthetase | BGC0000398 | Orfamide\_biosynthetic\_gene\_cluster | 26 | 96.1613532856 | 347 | 5.7e-95 |
| CAE02631.1 | surfactin\_synthetase\_B | BGC0000433 | Surfactin\_biosynthetic\_gene\_cluster | 25 | 102.212101496 | 347 | 5.7e-95 |
| AGM14933.1 | xantholysin\_synthetase\_B | BGC0000463 | Xantholysin\_biosynthetic\_gene\_cluster | 26 | 97.8529603123 | 346 | 1.3e-94 |
| AHZ34241.1 | CipD | BGC0001389 | Cichopeptin\_biosynthetic\_gene\_cluster | 26 | 99.544567339 | 344 | 4.9e-94 |
| AEG64698.1 | LpmD | BGC0000379 | Laspartomycin\_biosynthetic\_gene\_cluster | 25 | 98.1132075472 | 343 | 1.4e-93 |
| CAG15011.1 | peptide\_synthetase,\_module\_4-6 | BGC0000441 | Teicoplanin\_biosynthetic\_gene\_cluster | 29 | 70.7221860768 | 342 | 3.1e-93 |
| CCJ67639.1 | TaaD | BGC0000447 | Tolaasin\_biosynthetic\_gene\_cluster | 25 | 98.6337020169 | 342 | 3.1e-93 |
| AFP87549.1 | NrpS | BGC0001135 | Bicornutin\_biosynthetic\_gene\_cluster | 26 | 98.1782693559 | 342 | 3.1e-93 |
| AHZ34242.1 | CipE | BGC0001389 | Cichopeptin\_biosynthetic\_gene\_cluster | 27 | 96.2914769031 | 341 | 4.1e-93 |
| CCJ67640.1 | TaaE | BGC0000447 | Tolaasin\_biosynthetic\_gene\_cluster | 25 | 100.0 | 341 | 7e-93 |
| CAE53352.1 | non-ribosomal\_peptide\_synthetase | BGC0000440 | Teicoplanin\_biosynthetic\_gene\_cluster | 29 | 70.7221860768 | 340 | 9.2e-93 |
| AGE25479.1 | putative\_non-ribosomal\_peptide\_synthetase | BGC0001208\_c2 | Poaeamide\_biosynthetic\_gene\_cluster | 26 | 102.667534157 | 340 | 1.2e-92 |
| BAC67536.1 | arthrofactin\_synthetase\_C | BGC0000305 | Arthrofactin\_biosynthetic\_gene\_cluster | 27 | 98.6337020169 | 339 | 2e-92 |
| CAM56770.1 |  | BGC0000354 | Friulimicin\_biosynthetic\_gene\_cluster | 26 | 90.6961613533 | 339 | 2.7e-92 |
| ABW17377.1 | PsoC | BGC0000411 | Putisolvin\_biosynthetic\_gene\_cluster | 26 | 96.8770331815 | 338 | 4.5e-92 |
| AED90002.1 | non-ribosomal\_peptide\_synthetase\_ThaA | BGC0000443 | Thanamycin\_biosynthetic\_gene\_cluster | 26 | 87.9635653871 | 335 | 2.9e-91 |
| AAO72425.1 | syringopeptin\_synthetase\_C | BGC0000438 | Syringopeptin\_biosynthetic\_gene\_cluster | 26 | 106.180871828 | 334 | 5e-91 |
| ACA09733.2 | fusaricidin\_synthetase | BGC0000358 | Fusaricidin\_biosynthetic\_gene\_cluster | 25 | 87.3129472999 | 334 | 5e-91 |
| AAZ03552.1 | McnC | BGC0000332 | Cyanopeptolin\_biosynthetic\_gene\_cluster | 26 | 88.0936890046 | 334 | 5e-91 |
| AAC06348.1 | bacitracin\_synthetase\_3 | BGC0000310 | Bacitracin\_biosynthetic\_gene\_cluster | 24 | 101.821730644 | 334 | 8.6e-91 |
| AAY37655.1 | Amino\_acid\_adenylation | BGC0000437 | Syringomycin\_biosynthetic\_gene\_cluster | 26 | 101.171112557 | 334 | 8.6e-91 |
| AAO23333.1 | NcpA | BGC0000397 | Nostocyclopeptide\_biosynthetic\_gene\_cluster | 26 | 78.7247885491 | 333 | 1.1e-90 |
| AHD05614.1 | putative\_non-ribosomal\_peptide\_ligase/\_polyketide\_synthase\_hybrid | BGC0001033 | Paenilamicin\_biosynthetic\_gene\_cluster | 26 | 69.2257644763 | 333 | 1.5e-90 |
| AEZ51520.1 | PmxE | BGC0001153 | Polymyxin\_biosynthetic\_gene\_cluster | 25 | 105.790500976 | 332 | 1.9e-90 |
| AIW82283.1 | PuwF | BGC0001125 | Puwainaphycins\_biosynthetic\_gene\_cluster | 25 | 93.6890045543 | 332 | 1.9e-90 |
| ACA97580.1 | PmxE | BGC0000408 | Polymyxin\_biosynthetic\_gene\_cluster | 24 | 106.701366298 | 332 | 1.9e-90 |
| ANY57892.1 | PenN | BGC0001372 | Penigequinolone\_biosynthetic\_gene\_cluster | 27 | 69.8113207547 | 332 | 1.9e-90 |
| AAY37647.1 | Amino\_acid\_adenylation | BGC0000437 | Syringomycin\_biosynthetic\_gene\_cluster | 26 | 101.301236174 | 332 | 2.5e-90 |
| AGM16413.1 | paenibacterin\_synthetase\_B | BGC0000400 | Paenibacterin\_biosynthetic\_gene\_cluster | 27 | 74.6909564086 | 332 | 2.5e-90 |
| AOA33123.1 | nonribosomal\_peptide\_synthetase | BGC0001346 | Bananamides\_biosynthetic\_gene\_cluster | 26 | 98.0481457385 | 332 | 2.5e-90 |
| AHZ34233.1 | CifB | BGC0000323 | Cichofactin\_biosynthetic\_gene\_cluster | 26 | 100.650618087 | 332 | 2.5e-90 |
| AEU11003.1 | NpnC | BGC0001029 | Nostophycin\_biosynthetic\_gene\_cluster | 26 | 75.992192583 | 332 | 3.3e-90 |
| CBJ90082.1 | Non\_Ribosomal\_peptide\_synthetase\_(-succinylbenzoate--CoA\_ligase) | BGC0001132 | Xenotetrapeptide\_biosynthetic\_gene\_cluster | 29 | 74.0403383214 | 331 | 5.6e-90 |
| AGZ03651.1 | SevB | BGC0000426 | Sevadicin\_biosynthetic\_gene\_cluster | 28 | 72.6089785296 | 331 | 5.6e-90 |
| BAH43765.1 | tyrocidine\_synthetase\_II | BGC0000452 | Tyrocidine\_biosynthetic\_gene\_cluster | 28 | 67.5992192583 | 330 | 1.2e-89 |
| AAY37654.1 | Amino\_acid\_adenylation | BGC0000437 | Syringomycin\_biosynthetic\_gene\_cluster | 26 | 100.260247235 | 329 | 1.6e-89 |
| AAY37653.1 | Amino\_acid\_adenylation | BGC0000437 | Syringomycin\_biosynthetic\_gene\_cluster | 26 | 97.7878985036 | 329 | 1.6e-89 |
| ABV56588.1 | KtzH | BGC0000378 | Kutznerides\_biosynthetic\_gene\_cluster | 28 | 72.0234222511 | 329 | 2.1e-89 |
| AAM80539.1 | StaA | BGC0000290 | A47934\_biosynthetic\_gene\_cluster | 29 | 66.0377358491 | 328 | 3.6e-89 |
| AGS77309.1 | NRPS\_modules\_4-6 | BGC0001178 | UK-68,597\_biosynthetic\_gene\_cluster | 29 | 71.2426805465 | 328 | 3.6e-89 |
| ABH06369.2 | MassC | BGC0000389 | Massetolide\_A\_biosynthetic\_gene\_cluster | 26 | 100.260247235 | 328 | 4.7e-89 |
| AIG79243.1 | Hypothetical\_protein | BGC0000419 | Ristomycin\_A\_biosynthetic\_gene\_cluster | 29 | 65.7774886142 | 327 | 6.1e-89 |
| AEH59100.1 | amino\_acid\_adenylation\_domain-containing\_protein/NRPS | BGC0000385 | Lysobactin\_biosynthetic\_gene\_cluster | 26 | 96.4216005205 | 327 | 6.1e-89 |
| AEA30274.1 | peptide\_synthetase | BGC0000429 | Skyllamycin\_biosynthetic\_gene\_cluster | 26 | 97.1372804164 | 327 | 8e-89 |
| ANQ43349.1 | non-ribosomal\_peptide\_synthetase | BGC0001347\_c2 | Poaeamide\_B\_biosynthetic\_gene\_cluster | 26 | 90.6961613533 | 326 | 1.4e-88 |
| AAY91421.3 | non-ribosomal\_peptide\_synthetase\_OfaC | BGC0000399 | Orfamide\_biosynthetic\_gene\_cluster | 26 | 96.2264150943 | 326 | 1.8e-88 |
| BAH43870.1 | putative\_linear\_pentadecapeptide\_gramicidin\_synthetase\_LgrB | BGC0000367 | Gramicidin\_biosynthetic\_gene\_cluster | 27 | 72.739102147 | 326 | 2.3e-88 |
| CAY48789.1 | putative\_non-ribosomal\_peptide\_synthetase | BGC0001312 | Viscosin\_biosynthetic\_gene\_cluster | 26 | 105.07482108 | 326 | 2.3e-88 |
| AIW82284.1 | PuwG | BGC0001125 | Puwainaphycins\_biosynthetic\_gene\_cluster | 26 | 80.0260247235 | 326 | 2.3e-88 |
| ACY06285.1 | non-ribosomal\_peptide\_synthetase | BGC0001042 | Sanglifehrin\_A\_biosynthetic\_gene\_cluster | 27 | 93.5588809369 | 325 | 3e-88 |
| AIE77057.1 | peptide\_synthetase | BGC0000418 | Ristocetin\_biosynthetic\_gene\_cluster | 29 | 65.9076122316 | 324 | 5.2e-88 |
| ACM68690.1 | AerG1 | BGC0000298 | Aeruginosin\_biosynthetic\_gene\_cluster | 28 | 70.1366297983 | 324 | 6.8e-88 |
| AIE77059.1 | peptide\_synthetase | BGC0000418 | Ristocetin\_biosynthetic\_gene\_cluster | 28 | 70.3968770332 | 324 | 8.9e-88 |
| AAO72424.1 | syringopeptin\_synthetase\_B | BGC0000438 | Syringopeptin\_biosynthetic\_gene\_cluster | 26 | 100.455432661 | 324 | 8.9e-88 |
| AHZ34239.1 | CipB | BGC0001389 | Cichopeptin\_biosynthetic\_gene\_cluster | 27 | 80.4814573845 | 324 | 8.9e-88 |
| ANQ43347.1 | non-ribosomal\_peptide\_synthetase | BGC0001347 | Poaeamide\_B\_biosynthetic\_gene\_cluster | 29 | 71.6981132075 | 324 | 8.9e-88 |
| AAO62586.1 | peptide\_sythetase | BGC0001016 | Microcystin\_biosynthetic\_gene\_cluster | 27 | 80.2212101496 | 323 | 1.2e-87 |
| AFH75320.1 | nonribosomal\_peptide\_synthetase | BGC0000425 | Sessilin\_biosynthetic\_gene\_cluster | 28 | 80.2862719584 | 321 | 4.4e-87 |
| CAG15009.1 | peptide\_synthetase,\_module\_1-2 | BGC0000441 | Teicoplanin\_biosynthetic\_gene\_cluster | 29 | 66.5582303188 | 321 | 5.8e-87 |
| CAA11795.1 | PCZA363.4 | BGC0000322 | Chloroeremomycin\_biosynthetic\_gene\_cluster | 28 | 70.201691607 | 321 | 5.8e-87 |
| CAE53350.1 | non-ribosomal\_peptide\_synthetase | BGC0000440 | Teicoplanin\_biosynthetic\_gene\_cluster | 29 | 66.5582303188 | 321 | 5.8e-87 |
| AAU34202.1 | mannopeptimycin\_peptide\_synthetase\_MppA | BGC0000388 | Mannopeptimycin\_biosynthetic\_gene\_cluster | 25 | 95.9661678595 | 320 | 9.8e-87 |
| CAM59606.1 | non-ribosomal\_peptide\_synthetase | BGC0000297 | Aeruginoside\_biosynthetic\_gene\_cluster | 24 | 97.8529603123 | 320 | 9.8e-87 |
| AAK81826.1 | peptide\_synthetase | BGC0000326 | Complestatin\_biosynthetic\_gene\_cluster | 27 | 69.5510735198 | 320 | 1.3e-86 |
| AJV88375.1 | MfnC | BGC0001214 | Marformycins\_biosynthetic\_gene\_cluster | 26 | 82.6935588809 | 319 | 1.7e-86 |
| BAH43766.1 | tyrocidine\_synthetase\_III | BGC0000452 | Tyrocidine\_biosynthetic\_gene\_cluster | 27 | 70.7872478855 | 319 | 1.7e-86 |
| BAB69700.1 | iturin\_A\_synthetase\_C | BGC0001098 | Iturin\_biosynthetic\_gene\_cluster | 28 | 71.9583604424 | 319 | 1.7e-86 |
| AHB82058.1 | non\_ribosomal\_peptide\_synthetase | BGC0001019 | Microsclerodermins\_biosynthetic\_gene\_cluster | 27 | 77.7488614183 | 319 | 2.2e-86 |
| BAH22764.1 | nonribosomal\_peptide\_synthetase | BGC0001018 | Micropeptin\_biosynthetic\_gene\_cluster | 24 | 96.1613532856 | 319 | 2.2e-86 |
| ABI26078.1 | OciB | BGC0000331 | Cyanopeptin\_biosynthetic\_gene\_cluster | 25 | 93.0383864671 | 319 | 2.9e-86 |
| AAO56328.1 | non-ribosomal\_peptide\_synthetase\_SyfA | BGC0000435 | Syringafactin\_biosynthetic\_gene\_cluster | 25 | 94.5998698764 | 318 | 3.7e-86 |
| CAD17793.1 | probable\_non\_ribosomal\_peptide\_synthetase\_protein | BGC0001363 | Ralsolamycin\_biosynthetic\_gene\_cluster | 25 | 100.910865322 | 318 | 3.7e-86 |
| KPN93064.1 | NupB | BGC0001416 | Nunapeptin\_/\_nunamycin\_biosynthetic\_gene\_cluster | 25 | 99.0240728692 | 318 | 3.7e-86 |
| CAM02313.1 | putative\_non-ribosomal\_peptide\_synthetase | BGC0000349 | Erythrochelin\_biosynthetic\_gene\_cluster | 27 | 68.7052700065 | 318 | 3.7e-86 |
| AAF17281.1 | NosD | BGC0001028 | Nostopeptolide\_biosynthetic\_gene\_cluster | 24 | 103.448275862 | 318 | 3.7e-86 |
| CAD91211.1 | putative\_non-ribosomal\_peptide\_synthetase,\_module\_7 | BGC0000289 | A40926\_biosynthetic\_gene\_cluster | 26 | 96.8119713728 | 318 | 4.9e-86 |
| AAM80537.1 | StaC | BGC0000290 | A47934\_biosynthetic\_gene\_cluster | 28 | 69.8763825634 | 318 | 4.9e-86 |
| AIG79241.1 | Hypothetical\_protein | BGC0000419 | Ristomycin\_A\_biosynthetic\_gene\_cluster | 28 | 70.7221860768 | 318 | 4.9e-86 |
| BAB69699.1 | iturin\_A\_synthetase\_B | BGC0001098 | Iturin\_biosynthetic\_gene\_cluster | 27 | 79.8959011061 | 317 | 6.4e-86 |
| ABQ96384.2 | fusaricidin\_synthetase | BGC0001152 | Fusaricidin\_biosynthetic\_gene\_cluster | 25 | 72.0884840599 | 317 | 8.3e-86 |
| AGS77307.1 | NRPS\_modules\_1-2 | BGC0001178 | UK-68,597\_biosynthetic\_gene\_cluster | 28 | 64.7364996747 | 317 | 1.1e-85 |
| ABW17376.1 | PsoB | BGC0000411 | Putisolvin\_biosynthetic\_gene\_cluster | 25 | 98.6987638256 | 317 | 1.1e-85 |
| ABH06368.2 | MassB | BGC0000389 | Massetolide\_A\_biosynthetic\_gene\_cluster | 25 | 102.342225114 | 317 | 1.1e-85 |
| AHZ34238.1 | CipA | BGC0001389 | Cichopeptin\_biosynthetic\_gene\_cluster | 30 | 63.500325309 | 316 | 1.4e-85 |
| AHD05677.1 | nonribosomal\_peptide\_ligase\_subunit | BGC0000402 | Paenilarvins\_biosynthetic\_gene\_cluster | 27 | 69.746258946 | 316 | 1.4e-85 |
| ABW17375.1 | PsoA | BGC0000411 | Putisolvin\_biosynthetic\_gene\_cluster | 27 | 74.1704619388 | 316 | 2.4e-85 |
| AAF08796.1 | MycB | BGC0001103 | Mycosubtilin\_biosynthetic\_gene\_cluster | 26 | 70.201691607 | 315 | 3.2e-85 |
| CAK15814.1 | putative\_non-ribosomal\_peptide\_synthetase,\_terminal\_component | BGC0000344 | Entolysin\_biosynthetic\_gene\_cluster | 25 | 95.836044242 | 314 | 5.4e-85 |
| ABV79986.1 | ApnB | BGC0000301 | Anabaenopeptin\_biosynthetic\_gene\_cluster | 26 | 69.8113207547 | 314 | 5.4e-85 |
| ALK27915.1 | non-ribosomal\_peptide\_synthase | BGC0001233 | Feglymycin\_biosynthetic\_gene\_cluster | 25 | 91.9323357189 | 314 | 9.2e-85 |
| AHB82071.1 | non\_ribosomal\_peptide\_synthetase | BGC0001231 | Microsclerodermins\_biosynthetic\_gene\_cluster | 28 | 67.4040338321 | 313 | 1.2e-84 |
| CBG75492.1 | putative\_NRPS/siderophore\_biosynthesis\_protein | BGC0000423 | Scabichelin\_biosynthetic\_gene\_cluster | 29 | 67.2739102147 | 313 | 1.6e-84 |
| AAO23334.1 | NcpB | BGC0000397 | Nostocyclopeptide\_biosynthetic\_gene\_cluster | 26 | 81.067013663 | 313 | 1.6e-84 |
| AEP18655.1 | WAPS2 | BGC0000461 | WAP-8294A2\_(lotilibcin)\_biosynthetic\_gene\_cluster | 26 | 89.0696161353 | 313 | 1.6e-84 |
| AHF21229.1 | TriE | BGC0000449 | Tridecaptin\_biosynthetic\_gene\_cluster | 28 | 73.6499674691 | 312 | 2e-84 |
| ABX37382.1 | amino\_acid\_adenylation\_domain\_protein | BGC0000984 | Delftibactin\_biosynthetic\_gene\_cluster | 27 | 83.5393623943 | 312 | 2e-84 |
| AAF99707.2 | syringopeptin\_synthetase | BGC0000438 | Syringopeptin\_biosynthetic\_gene\_cluster | 27 | 84.3201040989 | 312 | 2e-84 |
| AGE11898.1 | nonribosomal\_peptide\_synthetase | BGC0000366 | Gobichelin\_biosynthetic\_gene\_cluster | 29 | 71.1125569291 | 312 | 2e-84 |
| CAJ34381.1 | NRPS\_protein | BGC0000445 | Thiocoraline\_biosynthetic\_gene\_cluster | 30 | 68.4450227716 | 312 | 2.7e-84 |
| AFH75329.1 | nonribosomal\_peptide\_synthetase | BGC0000398 | Orfamide\_biosynthetic\_gene\_cluster | 28 | 72.6089785296 | 312 | 3.5e-84 |
| CAD29797.1 | peptide\_synthetase | BGC0001015 | Microcystin\_biosynthetic\_gene\_cluster | 26 | 84.2550422902 | 311 | 4.6e-84 |
| AAK81824.1 | peptide\_synthetase | BGC0000326 | Complestatin\_biosynthetic\_gene\_cluster | 28 | 66.8835393624 | 311 | 7.8e-84 |
| CAB38518.1 | CDA\_peptide\_synthetase\_I | BGC0000315 | Calcium-dependent\_antibiotic\_biosynthetic\_gene\_cluster | 24 | 103.838646714 | 310 | 1e-83 |
| AAY91420.2 | non-ribosomal\_peptide\_synthetase\_OfaB | BGC0000399 | Orfamide\_biosynthetic\_gene\_cluster | 26 | 99.8048145738 | 310 | 1.3e-83 |
| CBZ42146.1 | putative\_non-ribosomal\_peptide\_synthetase | BGC0001117 | Himastatin\_biosynthetic\_gene\_cluster | 28 | 72.4137931034 | 310 | 1.3e-83 |
| AGE26255.1 | putative\_non-ribosomal\_peptide\_synthetase | BGC0001208 | Poaeamide\_biosynthetic\_gene\_cluster | 28 | 72.2186076773 | 310 | 1.3e-83 |
| CAD91220.1 | putative\_non-ribosomal\_peptide\_synthetase,\_modules\_1-2 | BGC0000289 | A40926\_biosynthetic\_gene\_cluster | 28 | 66.1027976578 | 309 | 1.7e-83 |
| CAJ96472.1 | Non-ribosomal\_peptide\_synthetase | BGC0000330 | Cupriachelin\_biosynthetic\_gene\_cluster | 27 | 79.6356538712 | 309 | 1.7e-83 |
| CAE53353.1 | non-ribosomal\_peptide\_synthetase | BGC0000440 | Teicoplanin\_biosynthetic\_gene\_cluster | 25 | 94.5348080677 | 309 | 2.3e-83 |
| AAF17280.1 | NosC | BGC0001028 | Nostopeptolide\_biosynthetic\_gene\_cluster | 24 | 103.253090436 | 308 | 3.9e-83 |
| CAC48360.1 | peptide\_synthetase | BGC0000311 | Balhimycin\_biosynthetic\_gene\_cluster | 28 | 65.0618087183 | 308 | 3.9e-83 |
| AHZ34232.1 | CifA | BGC0000323 | Cichofactin\_biosynthetic\_gene\_cluster | 27 | 72.1535458686 | 308 | 5e-83 |
| BAH22763.1 | nonribosomal\_peptide\_synthetase | BGC0001018 | Micropeptin\_biosynthetic\_gene\_cluster | 25 | 72.3487312947 | 307 | 6.6e-83 |
| CAG15012.1 | peptide\_synthetase,\_module\_7 | BGC0000441 | Teicoplanin\_biosynthetic\_gene\_cluster | 25 | 94.5348080677 | 307 | 8.6e-83 |
| AAZ03551.1 | McnB | BGC0000332 | Cyanopeptolin\_biosynthetic\_gene\_cluster | 25 | 72.4137931034 | 307 | 1.1e-82 |
| AEZ51516.1 | PmxA | BGC0001153 | Polymyxin\_biosynthetic\_gene\_cluster | 35 | 43.65647365 | 306 | 1.5e-82 |
| AFJ23825.1 | WLIP\_synthetase\_B | BGC0000462\_c2 | WLIP\_biosynthetic\_gene\_cluster | 28 | 71.6981132075 | 305 | 2.5e-82 |
| AAD44233.1 | PstA | BGC0000362 | Glycopeptidolipid\_biosynthetic\_gene\_cluster | 27 | 71.9583604424 | 305 | 3.3e-82 |
| ABS74206.1 | FenD | BGC0001095 | Fengycin\_biosynthetic\_gene\_cluster | 27 | 71.4378659727 | 305 | 3.3e-82 |
| AIW82285.1 | PuwH | BGC0001125 | Puwainaphycins\_biosynthetic\_gene\_cluster | 27 | 71.6330513988 | 304 | 5.6e-82 |
| AHH53508.1 | non-ribosomal\_peptide\_synthetase | BGC0000439 | Taromycin\_biosynthetic\_gene\_cluster | 25 | 92.7130774236 | 304 | 7.3e-82 |
| AEI58866.1 | peptide\_synthetase | BGC0000455 | Vancomycin\_biosynthetic\_gene\_cluster | 27 | 70.5270006506 | 304 | 7.3e-82 |
| AAK81827.1 | peptide\_synthetase | BGC0000326 | Complestatin\_biosynthetic\_gene\_cluster | 25 | 92.5178919974 | 303 | 1.6e-81 |
| ACA97576.1 | PmxA | BGC0000408 | Polymyxin\_biosynthetic\_gene\_cluster | 33 | 44.567338972 | 302 | 2.1e-81 |
| ANQ43348.1 | non-ribosomal\_peptide\_synthetase | BGC0001347\_c2 | Poaeamide\_B\_biosynthetic\_gene\_cluster | 25 | 102.732595966 | 302 | 2.1e-81 |
| CAB38517.1 | CDA\_peptide\_synthetase\_II | BGC0000315 | Calcium-dependent\_antibiotic\_biosynthetic\_gene\_cluster | 24 | 103.122966818 | 302 | 2.8e-81 |
| AAF15891.2 | NosA | BGC0001028 | Nostopeptolide\_biosynthetic\_gene\_cluster | 24 | 99.8048145738 | 302 | 2.8e-81 |
| AAM80536.1 | StaD | BGC0000290 | A47934\_biosynthetic\_gene\_cluster | 25 | 94.5348080677 | 302 | 2.8e-81 |
| AKC91849.1 | nonribosomal\_peptide\_synthetase | BGC0001414 | Griselimycin\_biosynthetic\_gene\_cluster | 27 | 66.5582303188 | 302 | 3.6e-81 |
| WP\_020635019.1 | hypothetical\_protein | BGC0001211 | Albachelin\_biosynthetic\_gene\_cluster | 27 | 71.828236825 | 301 | 6.2e-81 |
| BAH43871.1 | truncated\_linear\_pentadecapeptide\_gramicidin\_synthetase\_LgrC | BGC0000367 | Gramicidin\_biosynthetic\_gene\_cluster | 26 | 72.6740403383 | 300 | 8e-81 |
| AFJ23826.1 | WLIP\_synthetase\_C | BGC0000462\_c2 | WLIP\_biosynthetic\_gene\_cluster | 25 | 95.2504879636 | 300 | 8e-81 |
| ADG27358.1 | peptide\_synthetase | BGC0000296 | Actinomycin\_biosynthetic\_gene\_cluster | 25 | 95.4456733897 | 300 | 8e-81 |
| ALK27916.1 | non-ribosomal\_peptide\_synthase | BGC0001233 | Feglymycin\_biosynthetic\_gene\_cluster | 25 | 92.38776838 | 300 | 1.4e-80 |
| AHZ34240.1 | CipC | BGC0001389 | Cichopeptin\_biosynthetic\_gene\_cluster | 29 | 71.7631750163 | 300 | 1.4e-80 |
| CAC48361.1 | peptide\_synthetase | BGC0000311 | Balhimycin\_biosynthetic\_gene\_cluster | 27 | 70.2667534157 | 299 | 1.8e-80 |
| AJV88376.1 | MfnD | BGC0001214 | Marformycins\_biosynthetic\_gene\_cluster | 28 | 71.6981132075 | 299 | 2.3e-80 |
| EFL06866.1 | predicted\_protein | BGC0000300 | Amychelin\_biosynthetic\_gene\_cluster | 25 | 87.768379961 | 298 | 4e-80 |
| CAD91212.1 | putative\_non-ribosomal\_peptide\_synthetase,\_modules\_4-6 | BGC0000289 | A40926\_biosynthetic\_gene\_cluster | 27 | 70.5270006506 | 298 | 4e-80 |
| BAC67535.1 | arthrofactin\_synthetase\_B | BGC0000305 | Arthrofactin\_biosynthetic\_gene\_cluster | 25 | 97.8529603123 | 298 | 4e-80 |
| ACZ55944.1 | non-ribosomal\_peptide\_synthetase | BGC0000302 | Anabaenopeptin\_biosynthetic\_gene\_cluster | 26 | 70.0065061809 | 298 | 5.2e-80 |
| AOA33122.1 | nonribosomal\_peptide\_synthetase | BGC0001346 | Bananamides\_biosynthetic\_gene\_cluster | 27 | 80.4814573845 | 298 | 5.2e-80 |
| AJM89735.1 | PmxA | BGC0001192 | Colistin\_biosynthetic\_gene\_cluster | 34 | 41.9648666233 | 298 | 5.2e-80 |
| AHD05618.1 | putative\_non-ribosomal\_peptide\_ligase\_domain\_protein | BGC0001033 | Paenilamicin\_biosynthetic\_gene\_cluster | 26 | 70.0065061809 | 298 | 5.2e-80 |
| AEO14744.1 | NdaB | BGC0000396 | Nodularin\_biosynthetic\_gene\_cluster | 27 | 67.3389720234 | 296 | 1.5e-79 |
| CCM44336.1 | putative\_nonribosomal\_peptide\_synthetase | BGC0001056 | Zeamine\_biosynthetic\_gene\_cluster | 28 | 66.1027976578 | 296 | 2e-79 |
| AHD05678.1 | nonribosomal\_peptide\_ligase\_subunit | BGC0000402 | Paenilarvins\_biosynthetic\_gene\_cluster | 24 | 96.3565387118 | 296 | 2e-79 |
| AEI58867.1 | peptide\_synthetase | BGC0000455 | Vancomycin\_biosynthetic\_gene\_cluster | 26 | 97.3975276513 | 295 | 3.4e-79 |
| BAB69698.1 | iturin\_A\_synthetase\_A | BGC0001098 | Iturin\_biosynthetic\_gene\_cluster | 24 | 90.2407286923 | 295 | 3.4e-79 |
| ACZ55942.1 | non-ribosomal\_peptide\_synthetase | BGC0000302 | Anabaenopeptin\_biosynthetic\_gene\_cluster | 26 | 68.5100845804 | 295 | 3.4e-79 |
| AET13879.1 | epichloenin\_A\_synthetase | BGC0001251 | Epichloenin\_A\_biosynthetic\_gene\_cluster | 25 | 91.2817176318 | 294 | 5.8e-79 |
| ACZ55945.1 | non-ribosomal\_peptide\_synthetase | BGC0000302 | Anabaenopeptin\_biosynthetic\_gene\_cluster | 27 | 65.7124268055 | 294 | 5.8e-79 |
| AFH75328.1 | nonribosomal\_peptide\_synthetase | BGC0000398 | Orfamide\_biosynthetic\_gene\_cluster | 28 | 72.0884840599 | 294 | 5.8e-79 |
| AIE77060.1 | peptide\_synthetase\_module\_7 | BGC0000418 | Ristocetin\_biosynthetic\_gene\_cluster | 26 | 83.2140533507 | 294 | 5.8e-79 |
| AFJ23819.1 | WLIP\_synthetase\_A | BGC0000462 | WLIP\_biosynthetic\_gene\_cluster | 28 | 74.4957709824 | 294 | 5.8e-79 |
| ADJ63842.1 | non-ribosomal\_peptide\_synthetase\_protein | BGC0000424 | Serobactins\_biosynthetic\_gene\_cluster | 28 | 61.5484710475 | 294 | 9.8e-79 |
| CDF96614.1 | Long-chain-fatty-acid--CoA\_ligase | BGC0001149 | Brabantamide\_biosynthetic\_gene\_cluster | 25 | 86.6623292128 | 293 | 1.3e-78 |
| ABV79987.1 | ApnC | BGC0000301 | Anabaenopeptin\_biosynthetic\_gene\_cluster | 26 | 69.746258946 | 293 | 1.3e-78 |
| ABL74936.1 | TlmX | BGC0001048 | Tallysomycin\_biosynthetic\_gene\_cluster | 25 | 95.7059206246 | 293 | 1.3e-78 |
| ABI26077.1 | OciA | BGC0000331 | Cyanopeptin\_biosynthetic\_gene\_cluster | 27 | 66.4931685101 | 292 | 2.2e-78 |
| AET13875.1 | epichloenin\_A\_synthetase | BGC0001250 | Epichloenin\_A\_biosynthetic\_gene\_cluster | 25 | 91.2817176318 | 292 | 2.9e-78 |
| ABC87508.1 | NRPS\_for\_pipecolate\_incorporation | BGC0001011 | Meridamycin\_biosynthetic\_gene\_cluster | 24 | 95.9011060507 | 292 | 3.7e-78 |
| ctg1\_orf19 |  | BGC0001013 | Meridamycin\_biosynthetic\_gene\_cluster | 24 | 95.9011060507 | 292 | 3.7e-78 |
| ALV82356.1 | CDA\_peptide\_synthetase\_I | BGC0001370 | Lipopeptide\_8D1-1\_/\_lipopeptide\_8D1-2\_biosynthetic\_gene\_cluster | 27 | 84.9056603774 | 292 | 3.7e-78 |
| AAU34203.1 | mannopeptimycin\_peptide\_synthetase\_MppB | BGC0000388 | Mannopeptimycin\_biosynthetic\_gene\_cluster | 27 | 73.5198438517 | 291 | 4.9e-78 |
| EWS95122.1 | hypothetical\_protein | BGC0000306 | Arylomycin\_biosynthetic\_gene\_cluster | 27 | 70.4619388419 | 291 | 4.9e-78 |
| CAA11794.1 | PCZA363.3 | BGC0000322 | Chloroeremomycin\_biosynthetic\_gene\_cluster | 27 | 65.3220559532 | 291 | 6.4e-78 |
| AGN74886.1 | nonribosomal\_peptide\_synthetase | BGC0000459 | Griseoviridin\_/\_viridogrisein\_biosynthetic\_gene\_cluster | 27 | 88.9394925179 | 290 | 8.3e-78 |
| AIG79240.1 | Hypothetical\_protein | BGC0000419 | Ristomycin\_A\_biosynthetic\_gene\_cluster | 25 | 93.8841899805 | 290 | 1.4e-77 |
| AHH53507.1 | non-ribosomal\_peptide\_synthetase | BGC0000439 | Taromycin\_biosynthetic\_gene\_cluster | 25 | 97.7878985036 | 289 | 1.9e-77 |
| AHH53506.1 | non-ribosomal\_peptide\_synthetase | BGC0000439 | Taromycin\_biosynthetic\_gene\_cluster | 25 | 93.1034482759 | 289 | 2.4e-77 |
| CAE02633.1 | surfactin\_synthetase\_C | BGC0000433 | Surfactin\_biosynthetic\_gene\_cluster | 26 | 70.9173715029 | 289 | 2.4e-77 |
| CAE02630.1 | surfactin\_synthetase\_A | BGC0000433 | Surfactin\_biosynthetic\_gene\_cluster | 24 | 93.9492517892 | 289 | 3.2e-77 |
| BAH43764.1 | tyrocidine\_synthetase\_I | BGC0000452 | Tyrocidine\_biosynthetic\_gene\_cluster | 33 | 40.8588158751 | 289 | 3.2e-77 |
| ABF87031.1 | non-ribosomal\_peptide\_synthetase/polyketide\_synthase | BGC0000393 | Myxoprincomide\_biosynthetic\_gene\_cluster | 26 | 90.761223162 | 289 | 3.2e-77 |
| CAA11796.1 | PCZA363.5 | BGC0000322 | Chloroeremomycin\_biosynthetic\_gene\_cluster | 25 | 96.9420949902 | 288 | 5.4e-77 |
| AAZ55899.1 | amino\_acid\_adenylation | BGC0000359 | Fuscachelin\_biosynthetic\_gene\_cluster | 27 | 65.7124268055 | 287 | 7e-77 |
| AGS77310.1 | NRPS\_module\_7 | BGC0001178 | UK-68,597\_biosynthetic\_gene\_cluster | 25 | 94.8601171113 | 287 | 9.2e-77 |
| AEI70245.1 | nonribosomal\_peptide\_synthetase\_NRPS | BGC0000401 | Paenibactin\_biosynthetic\_gene\_cluster | 25 | 100.52049447 | 287 | 9.2e-77 |
| CCJ67636.1 | TaaA | BGC0000447 | Tolaasin\_biosynthetic\_gene\_cluster | 27 | 72.4137931034 | 287 | 9.2e-77 |
| ABS74207.1 | FenC | BGC0001095 | Fengycin\_biosynthetic\_gene\_cluster | 24 | 95.7059206246 | 286 | 1.6e-76 |
| AAO62588.1 | peptide\_sythetase | BGC0001016 | Microcystin\_biosynthetic\_gene\_cluster | 26 | 71.6981132075 | 286 | 1.6e-76 |
| ABS74180.1 | BmyB | BGC0001090 | Bacillomycin\_biosynthetic\_gene\_cluster | 26 | 79.7657774886 | 286 | 1.6e-76 |
| CAK15815.1 | putative\_non\_ribosomal\_peptide\_synthetase | BGC0000344 | Entolysin\_biosynthetic\_gene\_cluster | 27 | 79.8959011061 | 286 | 2e-76 |
| KFH48607.1 | N-like\_protein | BGC0000317 | Cephalosporin\_biosynthetic\_gene\_cluster | 25 | 81.1971372804 | 285 | 3.5e-76 |
| ABD65958.1 | nonribosomal\_peptide\_synthetase | BGC0000341 | Enduracidin\_biosynthetic\_gene\_cluster | 27 | 80.611581002 | 285 | 3.5e-76 |
| AIW82277.1 | PuwA | BGC0001125 | Puwainaphycins\_biosynthetic\_gene\_cluster | 27 | 70.0065061809 | 285 | 3.5e-76 |
| ABR67744.1 | CmnA | BGC0000316 | Capreomycin\_biosynthetic\_gene\_cluster | 26 | 79.2452830189 | 285 | 4.6e-76 |
| ACZ55946.1 | non-ribosomal\_peptide\_synthetase | BGC0000302 | Anabaenopeptin\_biosynthetic\_gene\_cluster | 30 | 44.9577098243 | 284 | 6e-76 |
| ABS74209.1 | FenA | BGC0001095 | Fengycin\_biosynthetic\_gene\_cluster | 24 | 88.7443070917 | 284 | 6e-76 |
| AGM14934.1 | xantholysin\_synthetase\_C | BGC0000463 | Xantholysin\_biosynthetic\_gene\_cluster | 27 | 71.1776187378 | 284 | 7.8e-76 |
| EAL92291.2 | nonribosomal\_peptide\_synthase\_GliP-like,\_putative | BGC0000372 | Hexadehydro-astechrome\_(HAS)\_biosynthetic\_gene\_cluster | 26 | 69.0305790501 | 284 | 7.8e-76 |
| EFE73312.1 | nonribosomal\_peptide\_synthetase | BGC0000431 | Stenothricin\_biosynthetic\_gene\_cluster | 28 | 72.2186076773 | 283 | 1.7e-75 |
| CCJ67637.1 | TaaB | BGC0000447 | Tolaasin\_biosynthetic\_gene\_cluster | 28 | 72.1535458686 | 283 | 1.7e-75 |
| AID65222.1 | putative\_aspartate\_racemase | BGC0000335 | Cystomanamides\_biosynthetic\_gene\_cluster | 29 | 63.6955107352 | 283 | 1.7e-75 |
| AGM16414.1 | paenibacterin\_synthetase\_C | BGC0000400 | Paenibacterin\_biosynthetic\_gene\_cluster | 27 | 74.1054001301 | 280 | 8.6e-75 |
| CBJ79916.1 | putative\_Ornithine\_racemase | BGC0001133 | Taxlllaid\_biosynthetic\_gene\_cluster | 23 | 96.4866623292 | 280 | 1.5e-74 |
| ABM21571.1 | CrpC | BGC0000975 | Cryptophycin\_biosynthetic\_gene\_cluster | 28 | 60.962914769 | 279 | 1.9e-74 |
| AFR69334.1 | nonribosomal\_peptide\_synthetase\_SpiDE1 | BGC0001045 | Spiruchostatin\_biosynthetic\_gene\_cluster | 27 | 68.119713728 | 279 | 1.9e-74 |
| ACM79806.1 | ZmaB | BGC0001059 | Zwittermycin\_A\_biosynthetic\_gene\_cluster | 27 | 60.4424202993 | 279 | 2.5e-74 |
| AIG79242.1 | Hypothetical\_protein | BGC0000419 | Ristomycin\_A\_biosynthetic\_gene\_cluster | 27 | 68.3799609629 | 279 | 3.3e-74 |
| AFJ14795.1 | PlpF | BGC0000403 | Pelgipeptin\_biosynthetic\_gene\_cluster | 30 | 51.0084580351 | 279 | 3.3e-74 |
| AAF00960.1 | McyA | BGC0001017 | Microcystin\_biosynthetic\_gene\_cluster | 26 | 71.1125569291 | 278 | 4.3e-74 |
| AAM80538.1 | StaB | BGC0000290 | A47934\_biosynthetic\_gene\_cluster | 27 | 70.2667534157 | 278 | 4.3e-74 |
| ABA70582.1 | alpha-aminoadypil-cysteinyl-valine\_synthetase | BGC0000404 | Penicillin\_biosynthetic\_gene\_cluster | 26 | 81.3272608979 | 278 | 4.3e-74 |
| BAC67534.2 | arthrofactin\_synthetase\_A | BGC0000305 | Arthrofactin\_biosynthetic\_gene\_cluster | 25 | 96.2914769031 | 278 | 5.6e-74 |
| CAC48362.1 | peptide\_synthetase | BGC0000311 | Balhimycin\_biosynthetic\_gene\_cluster | 25 | 96.5517241379 | 278 | 5.6e-74 |
| AAY93356.2 | non-ribosomal\_peptide\_synthetase\_PvdI | BGC0000413 | Pyoverdine\_biosynthetic\_gene\_cluster | 26 | 70.9173715029 | 277 | 7.3e-74 |
| ABR12615.1 | ACV\_synthetase | BGC0000405 | Penicillin\_biosynthetic\_gene\_cluster | 26 | 81.3272608979 | 277 | 9.5e-74 |
| CCA29203.1 | non-ribosomal\_peptide\_synthetase/polyketide\_synthase | BGC0000955 | Althiomycin\_biosynthetic\_gene\_cluster | 26 | 71.6981132075 | 277 | 1.2e-73 |
| AAG02355.1 | peptide\_synthetase\_NRPS9-8 | BGC0000963 | Bleomycin\_biosynthetic\_gene\_cluster | 32 | 43.9817826936 | 276 | 1.6e-73 |
| AAC06347.1 | bacitracin\_synthetase\_2 | BGC0000310 | Bacitracin\_biosynthetic\_gene\_cluster | 26 | 71.6330513988 | 276 | 1.6e-73 |
| CAB15186.3 | siderophore\_2,3-dihydroxybenzoate-glycine-threonine\_trimeric\_ester\_bacillibactin\_synthetase | BGC0000309 | Bacillibactin\_biosynthetic\_gene\_cluster | 25 | 77.1633051399 | 276 | 1.6e-73 |
| ADA69239.2 | trans-AT\_hybrid\_polyketide\_synthase-NRPS | BGC0001071 | Nosperin\_biosynthetic\_gene\_cluster | 28 | 62.6545217957 | 276 | 2.1e-73 |
| AEG64697.1 | LpmC | BGC0000379 | Laspartomycin\_biosynthetic\_gene\_cluster | 25 | 95.380611581 | 276 | 2.1e-73 |
| EFL06865.1 | hypothetical\_protein | BGC0000300 | Amychelin\_biosynthetic\_gene\_cluster | 24 | 96.5517241379 | 275 | 4.7e-73 |
| BAH22762.1 | nonribosomal\_peptide\_synthetase | BGC0001018 | Micropeptin\_biosynthetic\_gene\_cluster | 27 | 64.0208197788 | 275 | 4.7e-73 |
| ABS74179.1 | BmyC | BGC0001090 | Bacillomycin\_biosynthetic\_gene\_cluster | 31 | 47.8854912167 | 275 | 4.7e-73 |
| AOA33121.1 | nonribosomal\_peptide\_synthetase | BGC0001346 | Bananamides\_biosynthetic\_gene\_cluster | 29 | 54.8471047495 | 274 | 6.2e-73 |
| AAZ03550.1 | McnA | BGC0000332 | Cyanopeptolin\_biosynthetic\_gene\_cluster | 30 | 51.1385816526 | 274 | 8.1e-73 |
| AAY91419.3 | non-ribosomal\_peptide\_synthetase\_OfaA | BGC0000399 | Orfamide\_biosynthetic\_gene\_cluster | 27 | 71.7631750163 | 273 | 1.8e-72 |
| AAX31557.1 | peptide\_synthetase\_1 | BGC0000336 | Daptomycin\_biosynthetic\_gene\_cluster | 25 | 97.2674040338 | 273 | 1.8e-72 |
| AGC45618.1 | non-ribosomal\_peptide\_synthetase | BGC0001394 | Phenalamide\_biosynthetic\_gene\_cluster | 27 | 68.5100845804 | 273 | 1.8e-72 |
| CAD91221.1 | putative\_non-ribosomal\_peptide\_synthetase,\_module\_3 | BGC0000289 | A40926\_biosynthetic\_gene\_cluster | 27 | 72.2186076773 | 272 | 2.3e-72 |
| ABS75232.1 | DhbF | BGC0001185 | Bacillibactin\_biosynthetic\_gene\_cluster | 25 | 77.358490566 | 272 | 2.3e-72 |
| ABV79985.1 | ApnA | BGC0000301 | Anabaenopeptin\_biosynthetic\_gene\_cluster | 26 | 66.3630448926 | 272 | 3.1e-72 |
| AGS77308.1 | NRPS\_module\_3 | BGC0001178 | UK-68,597\_biosynthetic\_gene\_cluster | 27 | 64.5413142485 | 272 | 4e-72 |
| BAH43869.1 | linear\_pentadecapeptide\_gramicidin\_synthetase\_LgrA | BGC0000367 | Gramicidin\_biosynthetic\_gene\_cluster | 28 | 55.6929082628 | 271 | 5.2e-72 |
| AAK81825.1 | peptide\_synthetase | BGC0000326 | Complestatin\_biosynthetic\_gene\_cluster | 26 | 68.5751463891 | 271 | 6.8e-72 |
| CCE28989.1 | non-ribosomal\_peptide\_synthetase | BGC0001365 | Clapurines\_biosynthetic\_gene\_cluster | 24 | 93.298633702 | 271 | 6.8e-72 |
| CDG17982.1 | Non-ribosomal\_peptide\_synthetase | BGC0000464 | Xenoamicins\_biosynthetic\_gene\_cluster | 27 | 67.8594664932 | 271 | 6.8e-72 |
| AIE77058.1 | peptide\_synthetase\_module\_3 | BGC0000418 | Ristocetin\_biosynthetic\_gene\_cluster | 26 | 69.6161353286 | 270 | 8.9e-72 |
| CAD29799.1 | microcystin\_synthetase | BGC0001015 | Microcystin\_biosynthetic\_gene\_cluster | 27 | 61.4183474301 | 270 | 1.2e-71 |
| AAT09805.1 | NocB | BGC0000395 | Nocardicin\_A\_biosynthetic\_gene\_cluster | 26 | 68.5100845804 | 270 | 1.2e-71 |
| AAU39361.1 | lichenysin\_synthase\_LchAC | BGC0000381 | Lichenysin\_biosynthetic\_gene\_cluster | 25 | 70.8523096942 | 270 | 1.5e-71 |
| ABS74181.1 | BmyA | BGC0001090 | Bacillomycin\_biosynthetic\_gene\_cluster | 25 | 67.7293428757 | 270 | 1.5e-71 |
| AEW31021.1 | plipastatin\_synthetase | BGC0000407 | Plipastatin\_biosynthetic\_gene\_cluster | 27 | 71.1776187378 | 269 | 2.6e-71 |
| AID65225.1 | nonribosomal\_peptide\_synthetase | BGC0000335 | Cystomanamides\_biosynthetic\_gene\_cluster | 27 | 61.6135328562 | 269 | 3.4e-71 |
| AAY93354.1 | non-ribosomal\_peptide\_synthetase\_PvdD | BGC0000413 | Pyoverdine\_biosynthetic\_gene\_cluster | 25 | 70.6571242681 | 269 | 3.4e-71 |
| ABS74205.1 | FenE | BGC0001095 | Fengycin\_biosynthetic\_gene\_cluster | 27 | 62.3292127521 | 268 | 4.4e-71 |
| CAQ34921.1 | nonribosomal\_peptide\_synthetase | BGC0000986 | Dkxanthene\_biosynthetic\_gene\_cluster | 26 | 73.9752765127 | 268 | 5.8e-71 |
| BAH22765.1 | nonribosomal\_peptide\_synthetase | BGC0001018 | Micropeptin\_biosynthetic\_gene\_cluster | 27 | 67.1437865973 | 268 | 5.8e-71 |
| CAY48788.1 | putative\_non-ribosomal\_peptide\_synthetase | BGC0001312 | Viscosin\_biosynthetic\_gene\_cluster | 28 | 62.9147690306 | 268 | 5.8e-71 |
| ADZ24999.1 | non-ribosomal\_peptide\_synthase | BGC0000380 | Leupyrrin\_biosynthetic\_gene\_cluster | 28 | 70.8523096942 | 268 | 5.8e-71 |
| ALD82526.1 | non-ribosomal\_peptide\_synthase | BGC0001212 | Nannocystin\_biosynthetic\_gene\_cluster | 28 | 58.6206896552 | 267 | 7.5e-71 |
| AEW31015.1 | plipastatin\_synthetase | BGC0000407 | Plipastatin\_biosynthetic\_gene\_cluster | 26 | 72.5439167209 | 267 | 1.3e-70 |
| CAQ43084.1 | non\_ribosomal\_polypeptide\_synthetase | BGC0000970 | Chondrochloren\_biosynthetic\_gene\_cluster | 27 | 66.1678594665 | 267 | 1.3e-70 |
| CAJ14039.1 | peptide\_synthetase\_PhsC | BGC0000406 | Phosphinothricin\_biosynthetic\_gene\_cluster | 26 | 68.7052700065 | 266 | 2.2e-70 |
| AAF00962.1 | McyC | BGC0001017 | Microcystin\_biosynthetic\_gene\_cluster | 26 | 68.4450227716 | 266 | 2.2e-70 |
| AAK57184.1 | MxaA | BGC0001022 | Myxalamid\_biosynthetic\_gene\_cluster | 27 | 68.8353936239 | 265 | 3.7e-70 |
| ABJ97436.1 | MerP | BGC0001012 | Meridamycin\_biosynthetic\_gene\_cluster | 24 | 84.059856864 | 265 | 3.7e-70 |
| ALG65318.1 | Cal18 | BGC0001297 | WS9326\_biosynthetic\_gene\_cluster | 27 | 63.044892648 | 265 | 3.7e-70 |
| AAT12283.1 | LtxA | BGC0000384 | Lyngbyatoxin\_biosynthetic\_gene\_cluster | 28 | 62.9798308393 | 265 | 3.7e-70 |
| AJW76711.1 | DsaI | BGC0001196 | Desotamide\_biosynthetic\_gene\_cluster | 29 | 53.6759921926 | 265 | 4.9e-70 |
| AHZ20774.1 | non-ribosomal\_peptide\_synthase | BGC0000369 | Hassallidins\_biosynthetic\_gene\_cluster | 26 | 69.5510735198 | 265 | 4.9e-70 |
| AHJ31215.1 | Long-chain-fatty-acid--CoA\_ligase | BGC0000430 | Spumigin\_biosynthetic\_gene\_cluster | 26 | 65.126870527 | 264 | 6.4e-70 |
| AAF86395.1 | FkbP | BGC0000994 | FK520\_biosynthetic\_gene\_cluster | 25 | 84.3201040989 | 264 | 8.3e-70 |
| BAF50711.1 | non\_ribosomal\_peptide\_synthetase\_for\_virginiamycin\_S | BGC0001116 | Virginiamycin\_biosynthetic\_gene\_cluster | 27 | 72.6740403383 | 264 | 1.1e-69 |
| BAP05597.1 | CalI | BGC0000967 | Calyculin\_biosynthetic\_gene\_cluster | 31 | 45.738451529 | 263 | 1.4e-69 |
| ACG60782.1 | NRPS(C/A/PCP/C/A/PCP) | BGC0001058 | Zorbamycin\_biosynthetic\_gene\_cluster | 34 | 41.0540013012 | 263 | 1.4e-69 |
| AAU39359.1 | lichenysin\_synthase\_LchAA | BGC0000381 | Lichenysin\_biosynthetic\_gene\_cluster | 24 | 86.141834743 | 263 | 1.9e-69 |
| AAF08797.1 | MycC | BGC0001103 | Mycosubtilin\_biosynthetic\_gene\_cluster | 29 | 49.1216655823 | 262 | 2.4e-69 |
| AGN74876.1 | nonribosomal\_peptide\_synthetase | BGC0000459 | Griseoviridin\_/\_viridogrisein\_biosynthetic\_gene\_cluster | 26 | 83.0188679245 | 260 | 9.2e-69 |
| AAV97877.1 | OnnI | BGC0001105 | Onnamide\_biosynthetic\_gene\_cluster | 26 | 65.3871177619 | 260 | 1.2e-68 |
| ABC36785.1 | peptide\_synthetase,\_putative | BGC0000964 | Burkholdac\_biosynthetic\_gene\_cluster | 27 | 83.5393623943 | 260 | 1.2e-68 |
| ALV86867.1 | Tlo21 | BGC0001406 | Telomycin\_biosynthetic\_gene\_cluster | 27 | 66.2979830839 | 260 | 1.2e-68 |
| ACM79812.1 | ZmaQ | BGC0001059 | Zwittermycin\_A\_biosynthetic\_gene\_cluster | 26 | 57.9050097593 | 260 | 1.6e-68 |
| AAS47564.1 | mixed\_type\_I\_polyketide\_synthase/nonribosomal\_peptide\_synthetase | BGC0001108 | Pederin\_biosynthetic\_gene\_cluster | 26 | 65.9076122316 | 259 | 2.7e-68 |
| ctg1\_orf6 |  | BGC0001109 | Pederin\_biosynthetic\_gene\_cluster | 26 | 65.9076122316 | 259 | 2.7e-68 |
| CAL80824.1 | NRPS\_module\_protein | BGC0000997 | Glidobactin\_biosynthetic\_gene\_cluster | 27 | 71.1776187378 | 259 | 3.5e-68 |
| ACM68684.1 | AerB | BGC0000298 | Aeruginosin\_biosynthetic\_gene\_cluster | 26 | 64.2810670137 | 259 | 3.5e-68 |
| AJW76709.1 | DsaG | BGC0001196 | Desotamide\_biosynthetic\_gene\_cluster | 29 | 56.6688353936 | 258 | 6e-68 |
| AEA30273.1 | peptide\_synthetase | BGC0000429 | Skyllamycin\_biosynthetic\_gene\_cluster | 25 | 78.0741704619 | 258 | 6e-68 |
| ABI22132.1 | putative\_non-ribosomal\_peptide\_synthetase | BGC0000422 | Saframycin\_A\_biosynthetic\_gene\_cluster | 28 | 70.0715679896 | 257 | 7.8e-68 |
| AAN85512.1 | nonribosomal\_peptide\_synthetase | BGC0001101 | Leinamycin\_biosynthetic\_gene\_cluster | 28 | 62.9147690306 | 257 | 7.8e-68 |
| AIW82282.1 | PuwE | BGC0001125 | Puwainaphycins\_biosynthetic\_gene\_cluster | 29 | 53.80611581 | 257 | 1.3e-67 |
| AEH41794.1 | HrmP | BGC0000374 | Hormaomycin\_biosynthetic\_gene\_cluster | 25 | 84.1249186727 | 256 | 1.7e-67 |
| CAJ88610.1 | putative\_non-ribosomal\_peptide\_synthetase | BGC0000327 | Congocidine\_biosynthetic\_gene\_cluster | 26 | 66.5582303188 | 256 | 1.7e-67 |
| AGZ03650.1 | SevA | BGC0000426 | Sevadicin\_biosynthetic\_gene\_cluster | 31 | 40.2732595966 | 256 | 2.3e-67 |
| AAN32981.1 | BarG | BGC0000962 | Barbamide\_biosynthetic\_gene\_cluster | 28 | 51.9843851659 | 255 | 3e-67 |
| AAU39360.1 | lichenysin\_synthase\_LchAB | BGC0000381 | Lichenysin\_biosynthetic\_gene\_cluster | 25 | 72.8041639558 | 255 | 3e-67 |
| AED90003.1 | non-ribosomal\_peptide\_synthetase\_ThaB | BGC0000443 | Thanamycin\_biosynthetic\_gene\_cluster | 24 | 82.8887443071 | 255 | 3e-67 |
| CCJ67648.1 | JagD | BGC0001127 | Jagaricin\_biosynthetic\_gene\_cluster | 26 | 70.9173715029 | 255 | 5.1e-67 |
| AHI59109.1 | locillomycin\_synthase\_B | BGC0001005 | Locillomycin\_biosynthetic\_gene\_cluster | 23 | 84.1899804815 | 254 | 6.6e-67 |
| ABI26079.1 | OciC | BGC0000331 | Cyanopeptin\_biosynthetic\_gene\_cluster | 30 | 47.2999349382 | 253 | 1.1e-66 |
| AAZ03554.1 | McnE | BGC0000332 | Cyanopeptolin\_biosynthetic\_gene\_cluster | 26 | 67.5341574496 | 253 | 1.1e-66 |
| ANI24100.1 | nonribosomal\_peptide\_synthetase | BGC0001235 | Antalid\_biosynthetic\_gene\_cluster | 29 | 45.9336369551 | 253 | 1.5e-66 |
| CCE88377.1 | non-ribosomal\_peptide\_synthetase/polyketide\_synthase | BGC0001034 | Pellasoren\_biosynthetic\_gene\_cluster | 27 | 62.5243981783 | 253 | 1.9e-66 |
| CAJ21198.2 | non-ribosomal\_peptide\_synthetase | BGC0000297 | Aeruginoside\_biosynthetic\_gene\_cluster | 25 | 65.126870527 | 252 | 2.5e-66 |
| ALV86868.1 | Tlo22 | BGC0001406 | Telomycin\_biosynthetic\_gene\_cluster | 27 | 66.753415745 | 252 | 3.3e-66 |
| AEC14347.1 | nonribosomal\_peptide\_synthetase | BGC0000377 | Koranimine\_biosynthetic\_gene\_cluster | 26 | 69.4209499024 | 252 | 4.3e-66 |
| AAC68816.1 | FK506\_peptide\_synthetase | BGC0000353 | FK506\_biosynthetic\_gene\_cluster | 25 | 85.5562784645 | 251 | 7.3e-66 |
| AHB82059.1 | non\_ribosomal\_peptide\_synthetase/polyketide\_synthase | BGC0001019 | Microsclerodermins\_biosynthetic\_gene\_cluster | 27 | 57.6447625244 | 250 | 9.5e-66 |
| AJM89738.1 | PmxE | BGC0001192 | Colistin\_biosynthetic\_gene\_cluster | 29 | 54.9772283669 | 250 | 1.2e-65 |
| ACN39727.1 | SibD | BGC0000428 | Sibiromycin\_biosynthetic\_gene\_cluster | 26 | 69.0956408588 | 250 | 1.2e-65 |
| AAG02349.1 | peptide\_synthetase\_NRPS11-10 | BGC0000963 | Bleomycin\_biosynthetic\_gene\_cluster | 25 | 86.7273910215 | 250 | 1.6e-65 |
| ABL74945.1 | TlmIV | BGC0001048 | Tallysomycin\_biosynthetic\_gene\_cluster | 25 | 92.1925829538 | 249 | 2.1e-65 |
| AKC91856.1 | nonribosomal\_peptide\_synthetase | BGC0001414 | Griselimycin\_biosynthetic\_gene\_cluster | 31 | 41.7696811971 | 249 | 2.1e-65 |
| AEA30272.1 | peptide\_synthetase | BGC0000429 | Skyllamycin\_biosynthetic\_gene\_cluster | 26 | 92.38776838 | 249 | 2.8e-65 |
| AAZ23075.1 | peptide\_synthetase | BGC0000291 | A54145\_biosynthetic\_gene\_cluster | 27 | 68.2498373455 | 249 | 2.8e-65 |
| KPN90369.1 | NunE | BGC0001416 | Nunapeptin\_/\_nunamycin\_biosynthetic\_gene\_cluster | 24 | 75.992192583 | 249 | 2.8e-65 |
| CAJ18237.2 | non-ribosomal\_peptide\_synthetase\_B | BGC0000354 | Friulimicin\_biosynthetic\_gene\_cluster | 27 | 72.0234222511 | 248 | 3.6e-65 |
| ACA97577.1 | PmxB | BGC0000408 | Polymyxin\_biosynthetic\_gene\_cluster | 32 | 45.5432661028 | 248 | 4.7e-65 |
| CBW45647.1 | PI\_synthetase\_3\_and\_4 | BGC0000952 | Pristinamycin\_biosynthetic\_gene\_cluster | 27 | 68.7703318152 | 248 | 4.7e-65 |
| ABA23460.1 | Amino\_acid\_adenylation | BGC0000427 | Shinorine\_biosynthetic\_gene\_cluster | 31 | 40.9889394925 | 248 | 6.2e-65 |
| BAE98155.1 | putative\_non-ribosomal\_peptide\_synthetase | BGC0000339 | Echinomycin\_biosynthetic\_gene\_cluster | 32 | 40.4684450228 | 247 | 1.1e-64 |
| ABD65956.1 | nonribosomal\_peptide\_synthetase | BGC0000341 | Enduracidin\_biosynthetic\_gene\_cluster | 25 | 88.7443070917 | 247 | 1.4e-64 |
| AAY93445.1 | non-ribosomal\_peptide\_synthetase\_PvdL | BGC0000413 | Pyoverdine\_biosynthetic\_gene\_cluster | 30 | 46.5191932336 | 245 | 3.1e-64 |
| AAK06804.1 | putative\_peptide\_synthase\_SimD6 | BGC0001072 | Simocyclinone\_biosynthetic\_gene\_cluster | 27 | 54.0663630449 | 245 | 3.1e-64 |
| AAG34184.1 | SimH | BGC0000835 | Simocyclinone\_biosynthetic\_gene\_cluster | 27 | 54.0663630449 | 245 | 4e-64 |
| AAL15600.1 | SimH | BGC0000270 | Simocyclinone\_biosynthetic\_gene\_cluster | 27 | 54.0663630449 | 245 | 4e-64 |
| CAQ71828.1 | non\_ribosomal\_peptide\_synthase,\_antibiotic\_synthesis;\_contains\_3\_condensation\_domains,\_2\_AMP-acid\_ligases\_II\_domains,\_2\_PP-binding,\_Phosphopantetheine\_attachment\_site | BGC0001189 | Taiwachelin\_biosynthetic\_gene\_cluster | 27 | 68.4450227716 | 245 | 4e-64 |
| AHI59110.1 | locillomycin\_synthase\_C | BGC0001005 | Locillomycin\_biosynthetic\_gene\_cluster | 26 | 56.2134027326 | 244 | 6.8e-64 |
| AJM89734.1 | PmxB | BGC0001192 | Colistin\_biosynthetic\_gene\_cluster | 32 | 41.7046193884 | 244 | 8.9e-64 |
| AAS98786.1 | JamO | BGC0001001 | Jamaicamide\_biosynthetic\_gene\_cluster | 25 | 60.8978529603 | 242 | 3.4e-63 |
| AID65224.1 | nonribosomal\_peptide\_synthetase | BGC0000335 | Cystomanamides\_biosynthetic\_gene\_cluster | 25 | 72.0884840599 | 242 | 4.4e-63 |
| AAG29780.1 | acyl-CoA\_synthetase | BGC0000833 | Coumermycin\_A1\_biosynthetic\_gene\_cluster | 30 | 40.5335068315 | 241 | 5.8e-63 |
| ALV86866.1 | Tlo20 | BGC0001406 | Telomycin\_biosynthetic\_gene\_cluster | 26 | 66.3630448926 | 241 | 5.8e-63 |
| AHZ20781.1 | non-ribosomal\_peptide\_synthase | BGC0000369 | Hassallidins\_biosynthetic\_gene\_cluster | 25 | 72.1535458686 | 240 | 9.9e-63 |
| ALV82384.1 | CDA\_peptide\_synthetase\_II | BGC0001370 | Lipopeptide\_8D1-1\_/\_lipopeptide\_8D1-2\_biosynthetic\_gene\_cluster | 26 | 70.201691607 | 240 | 9.9e-63 |
| WP\_051700111.1 | non-ribosomal\_peptide\_synthetase | BGC0001368 | Tambromycin\_biosynthetic\_gene\_cluster | 27 | 67.4690956409 | 240 | 1.3e-62 |
| AAN65224.1 | peptide\_synthetase-like\_protein | BGC0000832 | Clorobiocin\_biosynthetic\_gene\_cluster | 30 | 40.4033832141 | 240 | 1.7e-62 |
| EWS95124.1 | hypothetical\_protein | BGC0000306 | Arylomycin\_biosynthetic\_gene\_cluster | 26 | 67.7293428757 | 239 | 2.2e-62 |
| AEG64696.1 | LpmB | BGC0000379 | Laspartomycin\_biosynthetic\_gene\_cluster | 26 | 69.4860117111 | 239 | 2.2e-62 |
| WP\_010369430.1 | Non-ribosomal\_peptide\_synthase | BGC0000314 | Bromoalterochromides\_biosynthetic\_gene\_cluster | 24 | 75.0813272609 | 239 | 2.2e-62 |
| EJK79843.1 | amino\_acid\_adenylation\_enzyme/thioester\_reductase\_family\_protein | BGC0000436 | Syringolin\_A\_biosynthetic\_gene\_cluster | 24 | 95.4456733897 | 238 | 3.8e-62 |
| ACM79810.1 | ZmaO | BGC0001059 | Zwittermycin\_A\_biosynthetic\_gene\_cluster | 24 | 65.8425504229 | 238 | 6.4e-62 |
| ctg1\_orf8 |  | BGC0001109 | Pederin\_biosynthetic\_gene\_cluster | 26 | 70.2667534157 | 237 | 8.4e-62 |
| AAS47562.1 | mixed\_type\_I\_polyketide\_synthase\_-\_peptide\_synthetase | BGC0001108 | Pederin\_biosynthetic\_gene\_cluster | 26 | 70.2667534157 | 237 | 8.4e-62 |
| AHD05617.1 | putative\_non-ribosomal\_peptide\_ligase\_domain\_protein | BGC0001033 | Paenilamicin\_biosynthetic\_gene\_cluster | 26 | 59.7918022121 | 237 | 8.4e-62 |
| AEZ51517.1 | PmxB | BGC0001153 | Polymyxin\_biosynthetic\_gene\_cluster | 31 | 41.8998048146 | 237 | 1.1e-61 |
| AFK57214.1 | DidC | BGC0000985 | Didemnin\_biosynthetic\_gene\_cluster | 28 | 56.9290826285 | 236 | 1.9e-61 |
| WP\_051700112.1 | non-ribosomal\_peptide\_synthetase | BGC0001368 | Tambromycin\_biosynthetic\_gene\_cluster | 25 | 68.4450227716 | 236 | 1.9e-61 |
| AGZ15458.1 | putative\_non-ribosomal\_peptide\_synthetase | BGC0001036 | Polyoxypeptin\_biosynthetic\_gene\_cluster | 29 | 47.104749512 | 236 | 2.4e-61 |
| AEW31022.1 | plipastatin\_synthetase | BGC0000407 | Plipastatin\_biosynthetic\_gene\_cluster | 31 | 43.2661027977 | 236 | 2.4e-61 |
| CBW45648.1 | PI\_synthetase\_2 | BGC0000952 | Pristinamycin\_biosynthetic\_gene\_cluster | 30 | 46.5191932336 | 236 | 2.4e-61 |
| CBJ79915.1 | putative\_Phenylalanine\_racemase\_(ATP-hydrolyzing) | BGC0001133 | Taxlllaid\_biosynthetic\_gene\_cluster | 26 | 53.6109303839 | 235 | 3.2e-61 |
| BAP05591.1 | CalC | BGC0000967 | Calyculin\_biosynthetic\_gene\_cluster | 29 | 44.4372153546 | 235 | 4.1e-61 |
| ABC94347.1 | vicibactin\_biosynthesis\_non-ribosomal\_peptide\_synthase\_protein | BGC0000457 | Vicibactin\_biosynthetic\_gene\_cluster | 28 | 55.3025374105 | 235 | 4.1e-61 |
| sln9 | nonribosomal\_peptide\_synthetase | BGC0001230 | Salinamides\_biosynthetic\_gene\_cluster | 32 | 41.7696811971 | 234 | 7.1e-61 |
| WP\_010369428.1 | phenylalanine\_racemase | BGC0000314 | Bromoalterochromides\_biosynthetic\_gene\_cluster | 28 | 54.4567338972 | 234 | 7.1e-61 |
| CAJ14037.1 | peptide\_synthetase\_PhsB | BGC0000406 | Phosphinothricin\_biosynthetic\_gene\_cluster | 27 | 63.1750162655 | 234 | 9.2e-61 |
| ABV56585.1 | KtzE | BGC0000378 | Kutznerides\_biosynthetic\_gene\_cluster | 30 | 40.4033832141 | 234 | 9.2e-61 |
| ABR67749.1 | CmnF | BGC0000316 | Capreomycin\_biosynthetic\_gene\_cluster | 27 | 63.044892648 | 233 | 1.2e-60 |
| AKA59447.1 | non-ribosomal\_peptide\_synthetase | BGC0001203 | Clarexpoxcin\_biosynthetic\_gene\_cluster | 28 | 56.9290826285 | 232 | 2.7e-60 |
| AAF67501.2 | NovH | BGC0000834 | Novobiocin\_biosynthetic\_gene\_cluster | 30 | 40.4684450228 | 232 | 3.5e-60 |
| CAN89638.1 | putative\_non-ribosomal\_peptide\_synthetase | BGC0001070 | Kirromycin\_biosynthetic\_gene\_cluster | 27 | 63.500325309 | 232 | 3.5e-60 |
| AKP45399.1 | CysK | BGC0001413 | Cystobactamide\_biosynthetic\_gene\_cluster | 24 | 82.4333116461 | 232 | 3.5e-60 |
| ABL74937.1 | TlmIX | BGC0001048 | Tallysomycin\_biosynthetic\_gene\_cluster | 30 | 45.738451529 | 232 | 3.5e-60 |
| ctg1\_orf00001 |  | BGC0000901 | Ferrichrome\_biosynthetic\_gene\_cluster | 32 | 31.0344827586 | 232 | 4.6e-60 |
| AGC09528.1 | NRPS | BGC0001183 | Lobophorin\_biosynthetic\_gene\_cluster | 29 | 44.9577098243 | 232 | 4.6e-60 |
| ACG60778.1 | NRPS(A/PCP) | BGC0001058 | Zorbamycin\_biosynthetic\_gene\_cluster | 31 | 41.11906311 | 232 | 4.6e-60 |
| AAN85501.1 | nonribosomal\_peptide\_synthetase | BGC0001101 | Leinamycin\_biosynthetic\_gene\_cluster | 30 | 42.3552374756 | 231 | 6e-60 |
| CDG12865.1 | non-ribosomal\_peptide\_synthetase | BGC0001415 | Althiomycin\_biosynthetic\_gene\_cluster | 26 | 59.0110605075 | 231 | 6e-60 |
| ABV79988.1 | ApnD | BGC0000301 | Anabaenopeptin\_biosynthetic\_gene\_cluster | 25 | 67.4690956409 | 230 | 1e-59 |
| AAZ55898.1 | amino\_acid\_adenylation | BGC0000359 | Fuscachelin\_biosynthetic\_gene\_cluster | 27 | 64.5413142485 | 230 | 1.3e-59 |
| ABD65960.1 | nonribosomal\_peptide\_synthetase | BGC0000341 | Enduracidin\_biosynthetic\_gene\_cluster | 30 | 41.574495771 | 230 | 1.7e-59 |
| AAC32048.1 | FxbC | BGC0000351 | Exochelin\_biosynthetic\_gene\_cluster | 27 | 58.425504229 | 229 | 2.3e-59 |
| CAE52339.1 | Polyketide\_non-ribosomal\_peptide\_synthase | BGC0001088 | Albicidin\_biosynthetic\_gene\_cluster | 26 | 70.0065061809 | 229 | 2.3e-59 |
| ACU71638.1 | amino\_acid\_adenylation\_domain\_protein | BGC0001154 | Cacibiocin\_biosynthetic\_gene\_cluster | 29 | 40.7937540664 | 229 | 2.3e-59 |
| AAZ23077.1 | peptide\_synthetase | BGC0000291 | A54145\_biosynthetic\_gene\_cluster | 27 | 72.4788549122 | 229 | 3e-59 |
| AGD80618.1 | non-ribosomal\_peptide\_synthetase | BGC0000394 | Naphthyridinomycin\_biosynthetic\_gene\_cluster | 26 | 68.3148991542 | 229 | 3e-59 |
| CAG15010.1 | peptide\_synthetase,\_module\_3 | BGC0000441 | Teicoplanin\_biosynthetic\_gene\_cluster | 31 | 40.6636304489 | 228 | 5.1e-59 |
| CAN89662.1 | putative\_non-ribosomal\_peptide\_synthetase | BGC0001070 | Kirromycin\_biosynthetic\_gene\_cluster | 29 | 41.4443721535 | 228 | 5.1e-59 |
| AHY86403.1 | non\_ribosomal\_peptide\_synthetase | BGC0000329 | Corpeptin\_biosynthetic\_gene\_cluster | 26 | 61.5484710475 | 228 | 6.6e-59 |
| AHB38497.1 | EpxD | BGC0000346 | Epoxomicin\_biosynthetic\_gene\_cluster | 28 | 54.3266102798 | 227 | 8.7e-59 |
| AIS24862.1 | Dst18 | BGC0001147 | Distamycin\_biosynthetic\_gene\_cluster | 30 | 40.5335068315 | 227 | 8.7e-59 |
| CCJ67646.1 | JagB | BGC0001127 | Jagaricin\_biosynthetic\_gene\_cluster | 27 | 55.9531554977 | 227 | 8.7e-59 |
| ALG65317.1 | Cal19 | BGC0001297 | WS9326\_biosynthetic\_gene\_cluster | 26 | 67.0136629798 | 227 | 1.1e-58 |
| CAJ46692.1 | non\_ribosomal\_peptide\_synthase | BGC0000969 | Chondramide\_biosynthetic\_gene\_cluster | 31 | 38.3864671438 | 227 | 1.5e-58 |
| AAC06346.1 | bacitracin\_synthetase\_1 | BGC0000310 | Bacitracin\_biosynthetic\_gene\_cluster | 24 | 52.9603122967 | 226 | 1.9e-58 |
| CAE53351.1 | non-ribosomal\_peptide\_synthetase | BGC0000440 | Teicoplanin\_biosynthetic\_gene\_cluster | 31 | 40.6636304489 | 226 | 2.5e-58 |
| ADL64235.1 | aureusimine\_non-ribosomal\_peptide\_synthetase | BGC0000308 | Aureusimine\_biosynthetic\_gene\_cluster | 26 | 58.1001951854 | 226 | 2.5e-58 |
| ACG60772.1 | NRPS(C/A/PCP/Cy/A/PCP/Cy) | BGC0001058 | Zorbamycin\_biosynthetic\_gene\_cluster | 27 | 61.1581001952 | 226 | 2.5e-58 |
| EFL06864.1 | non-ribosomal\_peptide\_synthetase | BGC0000300 | Amychelin\_biosynthetic\_gene\_cluster | 30 | 42.9407937541 | 225 | 3.3e-58 |
| CBW45749.1 | hybrid\_PKS/NRPS\_(PKSI) | BGC0000952\_c3 | Pristinamycin\_biosynthetic\_gene\_cluster | 26 | 64.0208197788 | 223 | 1.6e-57 |
| BAP16693.1 | nonribosomal\_peptide\_synthetase | BGC0000376 | JBIR-34,\_JBIR-35\_biosynthetic\_gene\_cluster | 31 | 40.0130123617 | 223 | 1.6e-57 |
| WP\_010369425.1 | ornithine\_racemase | BGC0000314 | Bromoalterochromides\_biosynthetic\_gene\_cluster | 28 | 43.5263500325 | 222 | 2.8e-57 |
| ERG36782.1 | Ornithine\_racemase | BGC0000299 | Alterochromides\_biosynthetic\_gene\_cluster | 28 | 43.5263500325 | 222 | 2.8e-57 |
| CAM56771.1 |  | BGC0000354 | Friulimicin\_biosynthetic\_gene\_cluster | 30 | 40.1431359792 | 222 | 3.6e-57 |
| AGZ15459.1 | putative\_non-ribosomal\_peptide\_synthetase | BGC0001036 | Polyoxypeptin\_biosynthetic\_gene\_cluster | 28 | 56.0832791152 | 222 | 4.7e-57 |
| ADY76664.1 | non-ribosomal\_peptide\_synthetase | BGC0000950 | Napsamycin\_biosynthetic\_gene\_cluster | 30 | 42.0949902407 | 220 | 1.8e-56 |
| BAP16697.1 | nonribosomal\_peptide\_synthetase | BGC0000376 | JBIR-34,\_JBIR-35\_biosynthetic\_gene\_cluster | 32 | 39.4274560833 | 219 | 2.4e-56 |
| ABL86391.1 | hybrid\_polyketide\_synthase\_and\_nonribosomal\_peptide\_synthetase | BGC0000999 | Heat-stable\_antifungal\_factor\_biosynthetic\_gene\_cluster | 25 | 66.3630448926 | 218 | 4e-56 |
| ABV56604.1 | adenylation\_domain\_protein | BGC0000378 | Kutznerides\_biosynthetic\_gene\_cluster | 30 | 39.7527651269 | 217 | 9e-56 |
| CAF05648.1 | TubC\_protein | BGC0001053 | Tubulysin\_biosynthetic\_gene\_cluster | 28 | 55.2374756018 | 217 | 1.2e-55 |
| AHB38515.1 | EpnG | BGC0000345 | Eponemycin\_biosynthetic\_gene\_cluster | 31 | 40.2081977879 | 216 | 2e-55 |
| ALG65313.1 | Cal23 | BGC0001297 | WS9326\_biosynthetic\_gene\_cluster | 29 | 41.574495771 | 216 | 2.6e-55 |
| CCC55927.1 | hypothetical\_protein | BGC0000973 | Collismycin\_A\_biosynthetic\_gene\_cluster | 34 | 29.4079375407 | 216 | 2.6e-55 |
| CAJ96468.1 | Non-ribosomal\_peptide\_synthetase\_modules\_and\_related\_proteins | BGC0000330 | Cupriachelin\_biosynthetic\_gene\_cluster | 29 | 44.2420299284 | 215 | 3.4e-55 |
| BAO84868.1 | putative\_non-ribosomal\_peptide\_synthetase | BGC0000414 | Quinocarcin\_biosynthetic\_gene\_cluster | 33 | 36.5647364997 | 214 | 7.6e-55 |
| CAJ45639.1 | vanchrobactin\_non\_ribosomal\_peptide\_synthetase | BGC0000454 | Vanchrobactin\_biosynthetic\_gene\_cluster | 24 | 72.6740403383 | 214 | 9.9e-55 |
| AHB82056.1 | non\_ribosomal\_peptide\_synthetase | BGC0001019 | Microsclerodermins\_biosynthetic\_gene\_cluster | 26 | 67.4040338321 | 214 | 9.9e-55 |
| CBA11570.1 | non-ribosomal\_peptide\_synthetase | BGC0001046 | Streptolydigin\_biosynthetic\_gene\_cluster | 34 | 34.0273259597 | 213 | 1.3e-54 |
| ADN26248.1 | peptide\_synthetase | BGC0000951 | Pacidamycin\_biosynthetic\_gene\_cluster | 29 | 42.1600520494 | 212 | 4.9e-54 |
| AFK57219.1 | DidH | BGC0000985 | Didemnin\_biosynthetic\_gene\_cluster | 29 | 45.0227716331 | 211 | 6.4e-54 |
| CBA63680.1 | nonribosomal\_peptide\_synthetase\_NRPS | BGC0000368 | Griseobactin\_biosynthetic\_gene\_cluster | 29 | 40.3383214053 | 211 | 6.4e-54 |
| AAG02364.1 | peptide\_synthetase\_NRPS2-1 | BGC0000963 | Bleomycin\_biosynthetic\_gene\_cluster | 26 | 64.1509433962 | 211 | 8.4e-54 |
| EOY45602.1 | non-ribosomal\_peptide\_synthetase | BGC0001168 | Livipeptin\_biosynthetic\_gene\_cluster | 30 | 37.410540013 | 211 | 8.4e-54 |
| CBL93718.1 | NRPS\_didomain\_PCP-C | BGC0000360 | GE81112\_biosynthetic\_gene\_cluster | 26 | 62.134027326 | 210 | 1.1e-53 |
| ADN26252.1 | peptide\_synthetase | BGC0000951 | Pacidamycin\_biosynthetic\_gene\_cluster | 31 | 39.037085231 | 210 | 1.1e-53 |
| CCA89328.1 | mixed\_trans-AT\_type\_I\_polyketide\_synthase/nonribosomal\_peptide\_synthetase | BGC0001111 | Rhizopodins\_biosynthetic\_gene\_cluster | 29 | 41.1841249187 | 210 | 1.4e-53 |
| AEA29644.1 | putative\_nonribosomal\_peptide\_synthetase\_and\_kinurenine\_monooxygenase | BGC0000409 | Porothramycin\_biosynthetic\_gene\_cluster | 27 | 64.0858815875 | 210 | 1.4e-53 |
| AGC09519.1 | AMP-dependent\_synthetase/ligase | BGC0001183 | Lobophorin\_biosynthetic\_gene\_cluster | 28 | 42.0949902407 | 210 | 1.4e-53 |
| CBL93723.1 | NRPS\_didomain\_A-PCP | BGC0000360 | GE81112\_biosynthetic\_gene\_cluster | 29 | 41.1841249187 | 210 | 1.4e-53 |
| EJK79842.1 | amino\_acid\_adenylation\_enzyme/thioester\_reductase\_family\_protein | BGC0000436 | Syringolin\_A\_biosynthetic\_gene\_cluster | 26 | 53.1554977228 | 210 | 1.9e-53 |
| AEO14743.1 | NdaA | BGC0000396 | Nodularin\_biosynthetic\_gene\_cluster | 32 | 37.0201691607 | 210 | 1.9e-53 |
| WP\_051700122.1 | non-ribosomal\_peptide\_synthetase | BGC0001368 | Tambromycin\_biosynthetic\_gene\_cluster | 29 | 42.6154847105 | 209 | 2.4e-53 |
| BAP82667.1 | non-ribosomal\_peptide\_synthetase\_A-domain\_containing\_protein | BGC0001148 | Pheganomycin\_biosynthetic\_gene\_cluster | 30 | 36.6297983084 | 209 | 3.2e-53 |
| AKA59436.1 | non-ribosomal\_peptide\_synthetase | BGC0001202 | Landepoxcin\_biosynthetic\_gene\_cluster | 26 | 66.753415745 | 209 | 3.2e-53 |
| AKP45395.1 | CysG | BGC0001413 | Cystobactamide\_biosynthetic\_gene\_cluster | 28 | 40.9238776838 | 209 | 3.2e-53 |
| AGN74885.1 | nonribosomal\_peptide\_synthetase | BGC0000459 | Griseoviridin\_/\_viridogrisein\_biosynthetic\_gene\_cluster | 27 | 59.1411841249 | 208 | 4.2e-53 |
| CCM44337.1 | putative\_nonribosomal\_peptide\_synthetase | BGC0001056 | Zeamine\_biosynthetic\_gene\_cluster | 31 | 38.3864671438 | 208 | 5.4e-53 |
| AHB82069.1 | non\_ribosomal\_peptide\_synthetase | BGC0001231 | Microsclerodermins\_biosynthetic\_gene\_cluster | 26 | 70.1366297983 | 208 | 7.1e-53 |
| CAD70195.1 | non-ribosomal\_peptide\_synthetase | BGC0001047 | Syringolin\_A\_biosynthetic\_gene\_cluster | 24 | 79.5055302537 | 207 | 1.2e-52 |
| CAD32904.2 | non-ribosomal\_peptide\_synthetase\_A | BGC0000354 | Friulimicin\_biosynthetic\_gene\_cluster | 27 | 43.4612882238 | 205 | 6e-52 |
| BAP27942.1 | nonribosomal\_peptide\_synthetase | BGC0001085 | Teleocidin\_B\_biosynthetic\_gene\_cluster | 33 | 28.7573194535 | 204 | 7.8e-52 |
| AFK57212.1 | DidA | BGC0000985 | Didemnin\_biosynthetic\_gene\_cluster | 24 | 85.2960312297 | 204 | 1e-51 |
| ALG65319.1 | Cal17 | BGC0001297 | WS9326\_biosynthetic\_gene\_cluster | 32 | 30.5790500976 | 204 | 1e-51 |
| AAL33757.1 | putative\_non-ribosomal\_peptide\_synthetase | BGC0000421 | Safracin\_biosynthetic\_gene\_cluster | 29 | 37.865972674 | 203 | 1.3e-51 |
| BAF50727.1 | hybrid\_polyketide\_synthase-non\_ribosomal\_peptide\_synthetase | BGC0001116 | Virginiamycin\_biosynthetic\_gene\_cluster | 30 | 39.492517892 | 203 | 1.3e-51 |
| AGN74898.1 | nonribosomal\_peptide\_synthetase | BGC0000459 | Griseoviridin\_/\_viridogrisein\_biosynthetic\_gene\_cluster | 30 | 33.9622641509 | 203 | 2.3e-51 |
| AEF16059.1 | non-ribosomal\_peptide\_synthetase | BGC0000953 | Amicetin\_biosynthetic\_gene\_cluster | 29 | 42.6805465192 | 202 | 3e-51 |
| ADY76667.1 | non-ribosomal\_peptide\_synthetase | BGC0000950 | Napsamycin\_biosynthetic\_gene\_cluster | 29 | 38.7117761874 | 201 | 5.1e-51 |
| AJV88377.1 | MfnE | BGC0001214 | Marformycins\_biosynthetic\_gene\_cluster | 35 | 28.7573194535 | 201 | 5.1e-51 |
| AGS06887.1 | mixed\_type\_I\_polyketide\_synthase/non-ribosomal\_peptide\_synthetase,\_PedF-like\_protein | BGC0001092\_c2 | Diaphorin\_biosynthetic\_gene\_cluster | 25 | 54.1964866623 | 201 | 6.6e-51 |
| ADN26251.1 | ATP-dependent\_adenylase | BGC0000951 | Pacidamycin\_biosynthetic\_gene\_cluster | 31 | 34.3526350033 | 201 | 8.7e-51 |
| ADC79642.1 | TamD | BGC0001052 | Tirandamycin\_biosynthetic\_gene\_cluster | 25 | 68.3799609629 | 200 | 1.5e-50 |
| CAE52334.1 | non-ribosomal\_peptide\_synthase | BGC0001088 | Albicidin\_biosynthetic\_gene\_cluster | 25 | 69.4860117111 | 200 | 1.9e-50 |
| ALD82525.1 | non-ribosomal\_peptide\_synthase | BGC0001212 | Nannocystin\_biosynthetic\_gene\_cluster | 31 | 29.2127521145 | 200 | 1.9e-50 |
| CCJ67647.1 | JagC | BGC0001127 | Jagaricin\_biosynthetic\_gene\_cluster | 30 | 41.0540013012 | 199 | 2.5e-50 |
| AJI44176.1 | nonribosomal\_peptide\_synthetase | BGC0001193 | Thiolutin\_biosynthetic\_gene\_cluster | 29 | 41.6395575797 | 198 | 4.3e-50 |
| ADF88265.1 | mixed\_nonribosomal\_peptide\_synthetase/\_polyketide\_synthase | BGC0000980 | Cylindrospermopsin\_biosynthetic\_gene\_cluster | 34 | 31.1646063761 | 198 | 5.6e-50 |
| ADF88262.1 | mixed\_nonribosomal\_peptide\_synthetase/\_polyketide\_synthase | BGC0000979 | Cylindrospermopsin\_biosynthetic\_gene\_cluster | 34 | 31.1646063761 | 198 | 5.6e-50 |
| AKA59440.1 | non-ribosomal\_peptide\_synthetase | BGC0001202 | Landepoxcin\_biosynthetic\_gene\_cluster | 29 | 40.0130123617 | 198 | 5.6e-50 |
| ADF88279.1 | mixed\_NRPS/PKS | BGC0000981 | Cylindrospermopsin\_biosynthetic\_gene\_cluster | 34 | 31.1646063761 | 198 | 5.6e-50 |
| AAC32047.1 | FxbB | BGC0000351 | Exochelin\_biosynthetic\_gene\_cluster | 28 | 41.9648666233 | 198 | 5.6e-50 |
| CAJ76292.1 | putative\_non-ribosomal\_peptide\_synthase | BGC0000972 | Colibactin\_biosynthetic\_gene\_cluster | 26 | 71.1776187378 | 198 | 7.3e-50 |
| ABX60161.1 | mixed\_NRPS/PKS | BGC0000978 | Cylindrospermopsin\_biosynthetic\_gene\_cluster | 33 | 31.1646063761 | 197 | 1.3e-49 |
| AAF19811.1 | MtaC | BGC0001024 | Myxothiazol\_biosynthetic\_gene\_cluster | 28 | 42.485361093 | 196 | 1.6e-49 |
| CAB53322.1 | putative\_peptide\_synthetase | BGC0000325 | Coelichelin\_biosynthetic\_gene\_cluster | 30 | 40.7937540664 | 196 | 1.6e-49 |
| AGN74892.1 | nonribosomal\_peptide\_synthetase/polyketide\_synthase\_hybrid\_protein | BGC0000459 | Griseoviridin\_/\_viridogrisein\_biosynthetic\_gene\_cluster | 30 | 35.0683148992 | 196 | 2.1e-49 |
| ABA59548.1 | Vlm2 | BGC0000453 | Valinomycin\_biosynthetic\_gene\_cluster | 26 | 56.6037735849 | 195 | 3.6e-49 |
| ADB92568.1 | CcbC | BGC0001225 | Celesticetin\_biosynthetic\_gene\_cluster | 29 | 34.2225113858 | 195 | 3.6e-49 |
| EDY47118.1 | N-(5-amino-5-carboxypentanoyl)-L-cysteinyl-D-\_valine\_synthase | BGC0000319 | Cephamycin\_C\_biosynthetic\_gene\_cluster | 28 | 43.1359791802 | 195 | 4.8e-49 |
| WP\_028678148.1 | non-ribosomal\_peptide\_synthetase | BGC0001228 | Retimycin\_biosynthetic\_gene\_cluster | 25 | 55.5627846454 | 195 | 4.8e-49 |
| BAP05589.1 | CalA | BGC0000967 | Calyculin\_biosynthetic\_gene\_cluster | 32 | 33.6369551074 | 194 | 8.1e-49 |
| AFD30954.1 | CrmA | BGC0000966 | Caerulomycin\_A\_biosynthetic\_gene\_cluster | 29 | 42.8106701366 | 194 | 8.1e-49 |
| CAD15508.1 | probable\_polyketide\_synthase\_protein | BGC0001014 | Micacocidin\_biosynthetic\_gene\_cluster | 34 | 28.2368249837 | 193 | 1.8e-48 |
| AHZ20784.1 | non-ribosomal\_peptide\_synthase | BGC0000369 | Hassallidins\_biosynthetic\_gene\_cluster | 29 | 38.5165907612 | 193 | 2.4e-48 |
| AFP73394.1 | FusA | BGC0001268 | Fusarin\_biosynthetic\_gene\_cluster | 23 | 62.6545217957 | 192 | 4e-48 |
| AGD80616.1 | amino\_acid\_adenylation\_domain-containing\_protein | BGC0000394 | Naphthyridinomycin\_biosynthetic\_gene\_cluster | 27 | 42.0949902407 | 191 | 5.3e-48 |
| ADY76684.1 | non-ribosomal\_peptide\_synthetase | BGC0000950 | Napsamycin\_biosynthetic\_gene\_cluster | 30 | 34.2875731945 | 191 | 5.3e-48 |
| AGN74895.1 | nonribosomal\_peptide\_synthetase/polyketide\_synthase\_hybrid\_protein | BGC0000459 | Griseoviridin\_/\_viridogrisein\_biosynthetic\_gene\_cluster | 28 | 39.6226415094 | 191 | 5.3e-48 |
| BAH33409.1 | putative\_non-ribosomal\_peptide\_synthetase | BGC0000371 | Heterobactin\_biosynthetic\_gene\_cluster | 32 | 40.2081977879 | 190 | 1.5e-47 |
| AFK57220.1 | DidI | BGC0000985 | Didemnin\_biosynthetic\_gene\_cluster | 29 | 41.3793103448 | 190 | 1.5e-47 |
| AET98905.1 | putative\_non-ribosomal\_peptide\_synthetase | BGC0000415 | Quinomycin\_biosynthetic\_gene\_cluster | 30 | 32.4007807417 | 190 | 1.5e-47 |
| AAL33756.1 | putative\_non-ribosomal\_peptide\_synthetase | BGC0000421 | Safracin\_biosynthetic\_gene\_cluster | 27 | 42.7456083279 | 190 | 2e-47 |
| CAG29031.1 | nonribosomal\_peptide\_synthetase\_(modules\_1\_and\_2) | BGC0001023 | Myxochromide\_biosynthetic\_gene\_cluster | 33 | 30.709173715 | 190 | 2e-47 |
| BAI63289.1 | putative\_non-ribosomal\_peptide\_synthetase | BGC0000434 | SW-163\_biosynthetic\_gene\_cluster | 30 | 32.7260897853 | 189 | 3.4e-47 |
| CBW45640.1 | PII\_peptide\_synthetase | BGC0000952 | Pristinamycin\_biosynthetic\_gene\_cluster | 28 | 39.3623942746 | 189 | 3.4e-47 |
| AIT38302.1 | LstE | BGC0000382 | Lipstatin\_biosynthetic\_gene\_cluster | 33 | 28.6922576448 | 188 | 4.5e-47 |
| AAT28740.1 | FUSS | BGC0000064 | Fusarin\_biosynthetic\_gene\_cluster | 27 | 40.0780741705 | 188 | 4.5e-47 |
| ABC34137.1 | peptide\_synthetase,\_putative | BGC0000961 | Bactobolin\_biosynthetic\_gene\_cluster | 31 | 34.3526350033 | 187 | 1.3e-46 |
| AAP92505.1 | nonribosomal\_peptide\_synthetase | BGC0000458 | Viomycin\_biosynthetic\_gene\_cluster | 30 | 34.6779440468 | 186 | 2.2e-46 |
| AAP92496.1 | nonribosomal\_peptide\_synthetase | BGC0000458 | Viomycin\_biosynthetic\_gene\_cluster | 29 | 40.4684450228 | 185 | 3.8e-46 |
| WP\_051700105.1 | non-ribosomal\_peptide\_synthetase | BGC0001368 | Tambromycin\_biosynthetic\_gene\_cluster | 29 | 40.9889394925 | 185 | 3.8e-46 |
| CAJ34375.1 | NRPS | BGC0000445 | Thiocoraline\_biosynthetic\_gene\_cluster | 25 | 53.9362394275 | 185 | 3.8e-46 |
| ACN69986.1 | proline\_adenyltransferase | BGC0000079 | Indanomycin\_biosynthetic\_gene\_cluster | 31 | 34.1574495771 | 185 | 3.8e-46 |
| BAP16689.1 | nonribosomal\_peptide\_synthetase | BGC0000376 | JBIR-34,\_JBIR-35\_biosynthetic\_gene\_cluster | 27 | 45.8685751464 | 184 | 1.1e-45 |
| CAJ76290.1 | putative\_non-ribosomal\_peptide\_synthase | BGC0000972 | Colibactin\_biosynthetic\_gene\_cluster | 29 | 37.4756018217 | 183 | 1.4e-45 |
| AHA12086.1 | amino\_acid\_adenyltransferase | BGC0001172 | Chlorizidine\_A\_biosynthetic\_gene\_cluster | 32 | 34.3526350033 | 183 | 1.4e-45 |
| BAH04161.1 | putative\_non-ribosomal\_peptide\_synthetase | BGC0000450 | Triostin\_A\_biosynthetic\_gene\_cluster | 30 | 32.5959661679 | 183 | 1.9e-45 |
| AHB38511.1 | EpnJ | BGC0000345 | Eponemycin\_biosynthetic\_gene\_cluster | 28 | 41.7046193884 | 183 | 2.4e-45 |
| ABX71111.1 | Lct28 | BGC0000238 | Lactonamycin\_biosynthetic\_gene\_cluster | 31 | 35.1984385166 | 181 | 9.3e-45 |
| ADZ45333.1 | NRPS/PKS\_hybrid | BGC0001020 | Muraymycin\_biosynthetic\_gene\_cluster | 27 | 38.7117761874 | 179 | 2.7e-44 |
| ABM21572.1 | CrpD | BGC0000975 | Cryptophycin\_biosynthetic\_gene\_cluster | 25 | 45.3480806766 | 178 | 4.6e-44 |
| BAE98156.1 | putative\_non-ribosomal\_peptide\_synthetase | BGC0000339 | Echinomycin\_biosynthetic\_gene\_cluster | 25 | 54.5868575146 | 177 | 1e-43 |
| CAL69597.1 | nonribosomal\_peptide\_synthase | BGC0001049 | Tenellin\_biosynthetic\_gene\_cluster | 29 | 41.8347430059 | 177 | 1e-43 |
| ADN43685.1 | DmbS | BGC0001136 | Desmethylbassianin\_biosynthetic\_gene\_cluster | 26 | 56.0182173064 | 176 | 1.8e-43 |
| ABL74940.1 | TlmVI | BGC0001048 | Tallysomycin\_biosynthetic\_gene\_cluster | 24 | 66.4281067014 | 175 | 3.9e-43 |
| CBK62746.1 |  | BGC0001115 | Thiomarinol\_biosynthetic\_gene\_cluster | 28 | 40.9238776838 | 175 | 5.1e-43 |
| AFR69331.1 | nonribosomal\_peptide\_synthetase\_SpiA | BGC0001045 | Spiruchostatin\_biosynthetic\_gene\_cluster | 26 | 43.2661027977 | 175 | 6.7e-43 |
| ADQ55476.1 | non-ribosomal\_peptide\_synthase\_dimodule | BGC0000350 | ET-743\_biosynthetic\_gene\_cluster | 25 | 46.1288223813 | 173 | 2.5e-42 |
| ADB02873.1 | AzicV | BGC0000202 | Azicemicin\_biosynthetic\_gene\_cluster | 29 | 33.8972023422 | 172 | 3.3e-42 |
| CAQ34914.1 | putative\_L-prolyl\_AMP-ligase | BGC0000986 | Dkxanthene\_biosynthetic\_gene\_cluster | 28 | 35.2635003253 | 172 | 4.3e-42 |
| AAD24881.1 | putative\_acyl-CoA\_synthetase | BGC0000127 | Pyoluteorin\_biosynthetic\_gene\_cluster | 29 | 34.873129473 | 171 | 9.6e-42 |
| XP\_002384042.1 | NRPS-like\_enzyme,\_putative | BGC0001234 | 4,4'-piperazine-2,5-diyldimethyl-bis-phenol\_biosynthetic\_gene | 26 | 40.2081977879 | 168 | 6.2e-41 |
| CBF80487.1 | hybrid\_PKS-NRPS\_(Eurofung) | BGC0000959 | Aspyridone\_biosynthetic\_gene\_cluster | 27 | 41.9648666233 | 167 | 1.4e-40 |
| AEC14346.1 | nonribosomal\_peptide\_synthetase | BGC0000377 | Koranimine\_biosynthetic\_gene\_cluster | 27 | 33.7020169161 | 165 | 5.3e-40 |
| AAV66110.2 | fusaridione\_A\_synthetase | BGC0000992 | Fusaridione\_A\_biosynthetic\_gene\_cluster | 28 | 40.9238776838 | 163 | 2.6e-39 |
| AGC24265.1 | PrlK | BGC0001038 | Pyralomicin\_biosynthetic\_gene\_cluster | 28 | 32.9863370202 | 162 | 4.5e-39 |
| ABV56594.1 | KtzN | BGC0000378 | Kutznerides\_biosynthetic\_gene\_cluster | 28 | 33.9622641509 | 161 | 5.8e-39 |
| ABX71145.1 | Lcz28 | BGC0000237 | Lactonamycin\_Z\_biosynthetic\_gene\_cluster | 29 | 35.5237475602 | 159 | 3.8e-38 |
| ERG36783.1 | phenylalanine\_racemase | BGC0000299 | Alterochromides\_biosynthetic\_gene\_cluster | 27 | 40.1431359792 | 159 | 3.8e-38 |
| AAC83656.1 | dihydroaeruginoic\_acid\_synthetase | BGC0000412 | Pyochelin\_biosynthetic\_gene\_cluster | 29 | 27.9115159401 | 158 | 8.4e-38 |
| EFY94493.1 | nonribosomal\_peptide\_synthase\_GliP-like,\_putative | BGC0000337 | Destruxins\_biosynthetic\_gene\_cluster | 25 | 42.4202992843 | 157 | 1.1e-37 |
| AAN32978.1 | BarD | BGC0000962 | Barbamide\_biosynthetic\_gene\_cluster | 25 | 35.5237475602 | 156 | 1.9e-37 |
| BAL15718.1 | NRPS | BGC0000432 | Streptothricin\_biosynthetic\_gene\_cluster | 29 | 35.8490566038 | 151 | 7.9e-36 |
| BAC76476.1 | multifunctional\_polyketide-peptide\_synthase\_LkcA | BGC0001100 | Lankacidin\_biosynthetic\_gene\_cluster | 29 | 39.492517892 | 147 | 1.5e-34 |
| AGC65517.1 | TtbB | BGC0001050 | Thalassospiramide\_biosynthetic\_gene\_cluster | 29 | 30.5790500976 | 146 | 2.5e-34 |
| ACY02015.1 | enterobactin\_synthetase\_component\_F | BGC0000343 | Enterobactin\_biosynthetic\_gene\_cluster | 24 | 49.1216655823 | 145 | 4.3e-34 |
| ABP57748.1 | DepD | BGC0000993 | FK228\_biosynthetic\_gene\_cluster | 25 | 45.9986987638 | 144 | 1.3e-33 |
| CAH55654.1 | putative\_L-prolyl-AMP\_ligase | BGC0000259 | Prodigiosin\_biosynthetic\_gene\_cluster | 27 | 33.8972023422 | 141 | 8.2e-33 |
| CCB53267.1 | non-ribosomal\_peptide\_synthetase | BGC0001393 | Lugdunin\_biosynthetic\_gene\_cluster | 21 | 58.6206896552 | 141 | 1.1e-32 |
| AAW03330.1 | CtaG | BGC0000982 | Cystothiazole\_A\_biosynthetic\_gene\_cluster | 31 | 27.3910214704 | 139 | 3.1e-32 |
| CCB53266.1 | non-ribosomal\_peptide\_synthetase | BGC0001393 | Lugdunin\_biosynthetic\_gene\_cluster | 23 | 38.5816525699 | 139 | 3.1e-32 |
| CAH55637.1 | putative\_L-prolyl-AMP\_ligase | BGC0000258 | Prodigiosin\_biosynthetic\_gene\_cluster | 26 | 33.7020169161 | 138 | 5.3e-32 |
| CBZ42143.1 | putative\_non-ribosomal\_peptide\_synthetase | BGC0001117 | Himastatin\_biosynthetic\_gene\_cluster | 27 | 42.2251138582 | 138 | 9e-32 |
| AAF19815.1 | MtaG | BGC0001024 | Myxothiazol\_biosynthetic\_gene\_cluster | 33 | 25.4391672088 | 134 | 1e-30 |
| CAD89778.1 | MelG\_protein | BGC0001010 | Melithiazol\_biosynthetic\_gene\_cluster | 29 | 28.1067013663 | 131 | 1.1e-29 |
| AFO85453.1 | non-ribosomal\_peptide\_synthetase | BGC0000391 | Methylpendolmycin/pendolmycin\_biosynthetic\_gene\_cluster | 30 | 20.8848405986 | 124 | 1e-27 |
| BAO84864.1 | putative\_non-ribosomal\_peptide\_synthetase | BGC0000414 | Quinocarcin\_biosynthetic\_gene\_cluster | 27 | 23.096942095 | 102 | 5.5e-21 |
| CAJ76289.1 | putative\_hybrid\_non-ribosomal\_peptide-polyketide\_synthetase | BGC0000972 | Colibactin\_biosynthetic\_gene\_cluster | 21 | 42.7456083279 | 98 | 6.1e-20 |
| BAG16281.1 | putative\_2,3-dihydroxybenzoate-AMP\_ligase | BGC0000632 | Brasilicardin\_A\_biosynthetic\_gene\_cluster | 23 | 35.7189329863 | 81 | 7.7e-15 |
| ADX66470.1 | ScnS0 | BGC0000108 | Natamycin\_biosynthetic\_gene\_cluster | 23 | 41.3793103448 | 77 | 1.1e-13 |
| AAW49319.1 | thaxtomin\_synthetase\_B | BGC0000444 | Thaxtomin\_biosynthetic\_gene\_cluster | 24 | 21.8607677293 | 70 | 2.3e-11 |
| AAR16521.1 | RimA | BGC0000138 | Rimocidin\_biosynthetic\_gene\_cluster | 25 | 20.3643461288 | 62 | 4.8e-09 |
| ABA59547.1 | Vlm1 | BGC0000453 | Valinomycin\_biosynthetic\_gene\_cluster | 22 | 20.4944697463 | 57 | 1.5e-07 |
